# Cyclobutane-1,3-Diacid (CBDA): A Semi-Rigid Building Block Prepared by [2+2] Photocyclization for Polymeric Materials

**DOI:** 10.1038/s41598-017-13983-z

**Published:** 2017-10-20

**Authors:** Zhihan Wang, Benjamin Miller, Micah Mabin, Rahul Shahni, Zijun D. Wang, Angel Ugrinov, Qianli R. Chu

**Affiliations:** 1 Department of Chemistry, University of North Dakota, Grand Forks, ND 58202 USA; 2 Department of Chemistry and Biochemistry, North Dakota State University, Fargo, ND 58102 USA

## Abstract

A previously overlooked building block, cyclobutane-1,3-diacid (CBDA), is introduced to materials synthesis due to its great potentials. As an example of CBDA, *α*-truxillic acid or 2,4-diphenylcyclobutane-1,3-dicarboxylic acid, was readily synthesized from commercially available *trans*-cinnamic acid. This CBDA showed outstanding stability both in sunlight and upon heating. While its two carboxylic acid groups can be readily utilized in connecting with other molecules to form new materials, the cyclobutane ring was able to tolerate acid and base treatments showing good chemical stability. A series of cyclobutane-containing polymers (CBPs), namely poly-*α*-truxillates, were obtained by condensation between *α*-truxillic acid and diols including ethylene glycol, 1,3-propanediol, 1,4-butanediol, 1,5-petanediol, and 1,6-hexanediol. The structures of these poly-*α*-truxillates were analyzed by NMR, FT-IR, and HRMS. Powder X-ray diffraction results of the poly-*α*-truxillates indicated that they are semi-crystalline materials. Preliminary thermal, chemical, and photochemical tests showed that the poly-*α*-truxillates exhibited comparable stabilities to PET.

## Introduction

Since the dawn of civilization, the development of new materials has been the driving force behind technological advancement, and in the modern era, few materials have had a broader impact than synthetic polymers. Herein, we suggest that cyclobutane-1,3-diacid (CBDA) should be included as a semi-rigid building block in the research efforts to synthesize polymers^1,2^ and other materials such as metal-organic frameworks (MOFs)^3,4^. As a case study of CBDA, we present the monomer, *trans*-2,4-diphenylcyclobutane-1,3-dicarboxylic acid (**CBDA-1**), which is also known as *α*-truxillic acid.

**Figure 1:**
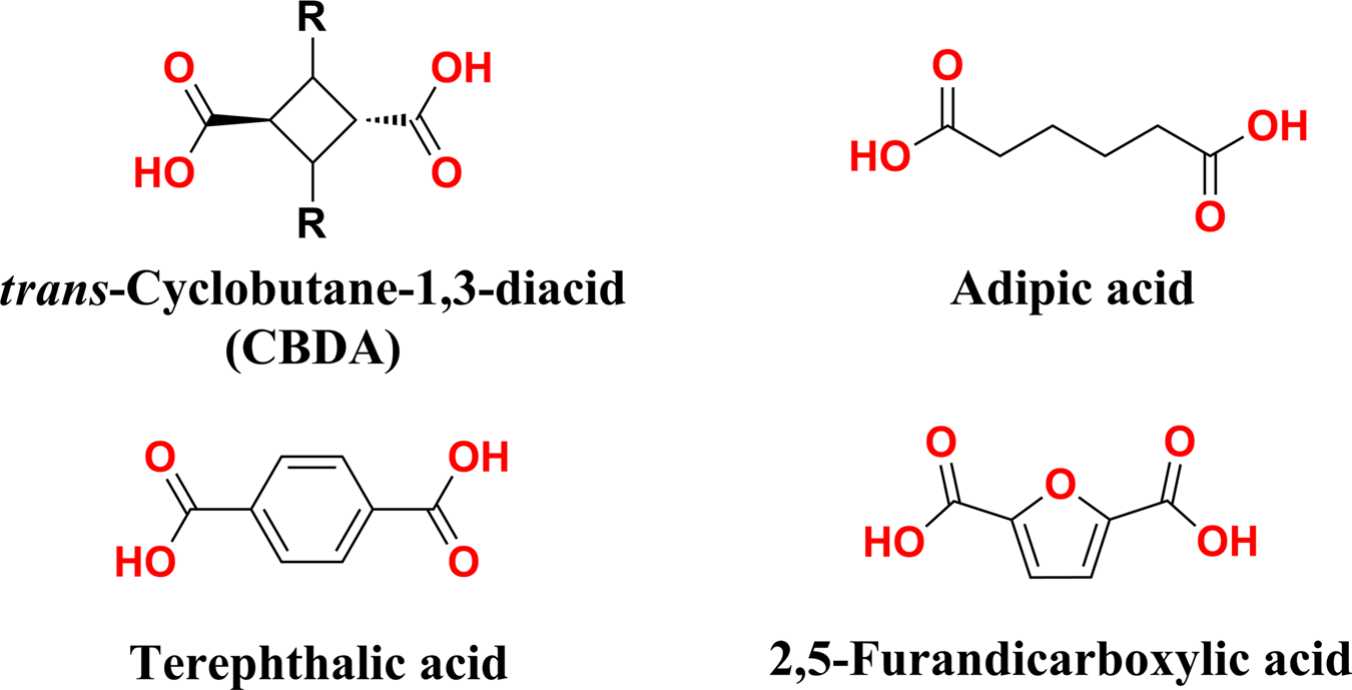
Diacid building blocks for materials synthesis.

Diacids similar to CBDA are widely used in modern materials (Fig. 1)^5–8^. A highly successful example is the aliphatic diacid, adipic acid, used to make Nylon 66^9,10^. Aromatic diacids have also found a variety of applications in materials. For instance, terephthalic acid, or benzene-1,4-dicarboxylic acid, is a chemical synthesized from a compound in crude oil^11,12^. It is a key building block in polyethylene terephthalate (PET), which is widely known for its use in plastic beverage bottles^13,14^. Researchers are currently trying to find a biomass-based diacid to serve as an alternative to terephthalic acid^15,16^. A prime candidate has been the furan-based building block 2,5-furandicarboxylic acid, which was named one of the top-12 value-added chemicals for “green” chemistry^17,18^. Compared to these classic diacids, which are either flexible aliphatic diacids and rigid aromatic diacids, CBDA represents a unique semi-flexible/semi-rigid building block in materials synthesis due to the presence of the small aliphatic ring with limited conformational freedom.

**Figure 2:**
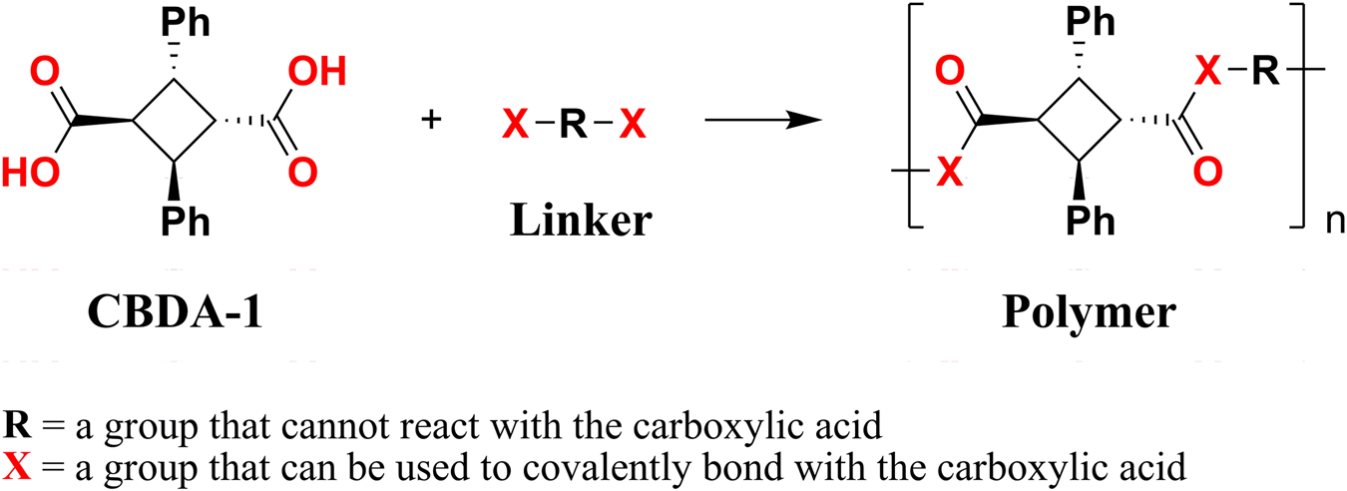
The strategy of using CBDA-1 as a building block in polymer synthesis.

Despite the prevalence of the cyclobutane unit in many natural products^19–25^ and synthetic drugs^26–35^, it is rarely seen in materials with industrial applications^36^, most likely because concern about its stability has discouraged experimentation with this promising building block. When compared to five- and six-membered carbon rings, four-membered carbon rings are indeed less stable. The ring strain energy of cyclobutane is about 20.1 and 26.2 kcal/mol higher than that of cyclopentane and cylcohexane, respectively^37^. However, thermal [2+2] cycloaddition is generally forbidden according to the Woodward-Hoffmann rules and it normally requires deep UV to cleave the four-membered carbon ring. Therefore, we hypothesized that cyclobutane has sufficient thermal and sunlight stability for many potential applications in materials. To test this hypothesis, we synthesized and examined the stability of **CBDA-1** and several of its polymeric derivatives. Incorporating **CBDA-1** into polymers requires a linker molecule capable of reacting with carboxylic acid (Fig. 2). A series of aliphatic diols were successfully used as linker molecules in combination with **CBDA-1** to create polyesters that showed excellent stability.

## Results

### Synthesis of CBDA-1

The method used to synthesize *trans*-2,4-diphenyl-1,3-cyclobutanedicarboxylic acid (**CBDA-1**) has been known for several decades^38^. It can be readily synthesized from commercially available *trans*-cinnamic acid^39–41^ *via* photodimerization in the solid-state^42–46^. This process can be completed in 8 hours in near quantitative yield without side-products allowing the obtained **CBDA-1** to be used in subsequent steps without further purification. The efficiency of the solvent-free photoreaction is presumably due to complementary π–π interactions between adjacent *trans*-cinnamic acid molecules, which are potentiated by head-to-tail (*α-*form) packing in the solid state. On one end of the molecule, phenyl groups act as weak electron donating groups while carboxylic acid groups on the opposite end function as weak electron accepting groups. The end result is that the flat, conjugated *trans*-cinnamic acid molecules are relatively polar and prefer a head-to-tail packing formation because it is lower in energy^42,47^. Quality single crystals of *trans*-cinnamic acid were obtained in a mixed solvent of ethyl acetate and acetonitrile (1:1). X-ray diffraction analysis confirmed its head-to-tail packing. We found that the head-to-tail packing could be easily obtained in a variety of solvents including acetonitrile, acetone, toluene, methanol, THF, and chloroform showing that the *α-*form is the dominant packing conformation for *trans*-cinnamic acid. Moreover, powder X-ray diffraction (PXRD) confirmed that the packing of commercial *trans*-cinnamic acid is the head-to-tail form because its powder pattern was nearly identical to that of the head-to-tail single crystal simulation (see Supplementary Figure S3). Consequently, the commercial *trans*-cinnamic acid powder can be used to produce the building block, **CBDA-1**, directly without recrystallization. It is also worthwhile to mention that only one of the five stereoisomers of the [2+2] head-to-tail dimers was produced because solid state photoreaction normally proceeds with minimal movement of atoms (see Figure S2 in the Supplementary).

### Thermal, chemical, and photochemical stability analyses

**Figure 3:**
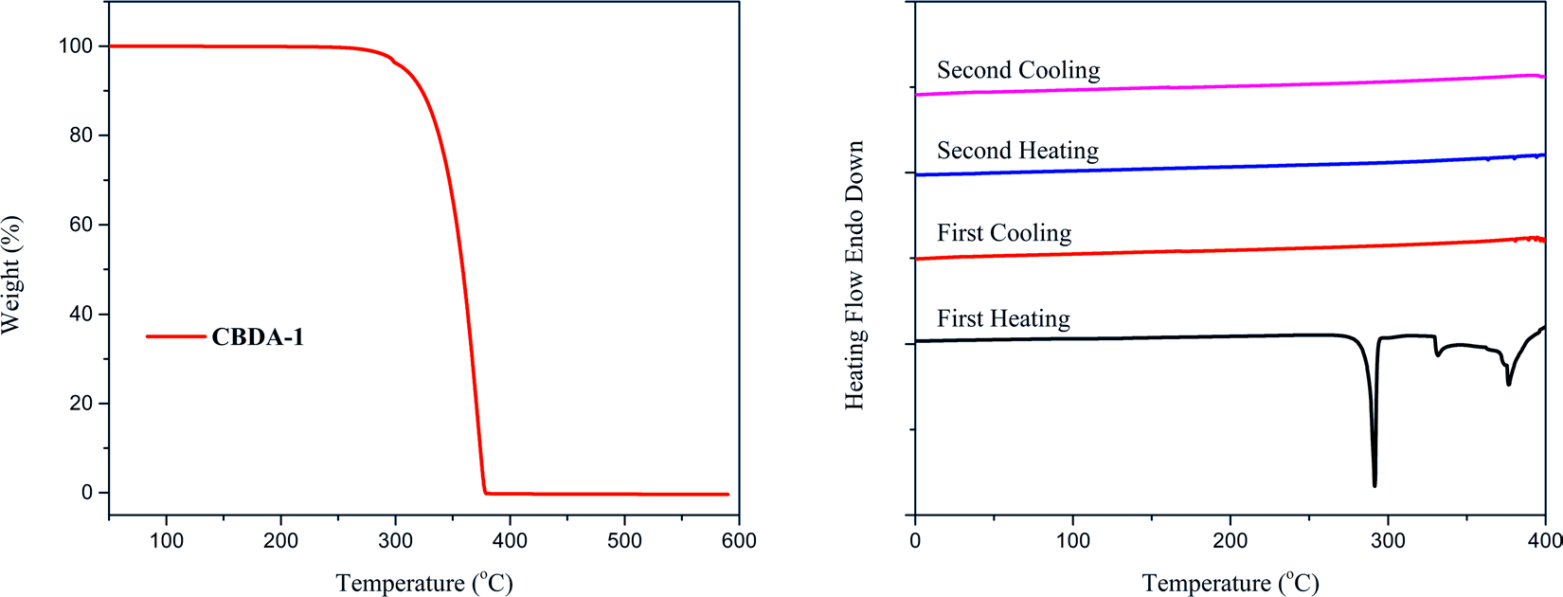
TGA (left) and DSC (right) of **CBDA-1**: TGA was recorded from 50 °C to 600 °C with a heating rate of 20 °C·min^−1^ under N_2_ atmosphere. DSC was recorded from 0 °C to 400 °C with a heating rate of 10 °C·min^−1^ under N_2_ atmosphere.

Despite the relatively straightforward synthetic pathway to create to **CBDA-1**, its utility as a building block in materials has been rarely studied. Our thermogravimetric analysis (TGA) and differential scanning calorimetry (DSC) study showed **CBDA-1** was stable at 250 °C and only lost 5% of its weight when the temperature is increased to 307 °C (Fig. 3). The weight lost in TGA below 330 °C may be due to evaporation because **CBDA-1** melts around 276 °C and it lost all weight at 378 °C showing its decomposition at high temperature. The DSC curve suggested **CBDA-1** started decomposing at 330 °C in the first heating process. After decomposition, DSC showed no change in the first cooling and second heating processes. The TGA and DSC curves showed that **CBDA-1** is thermally stable enough to be used as a building block in materials. The results are reasonable because [2+2] cycloaddition is generally photochemically allowed, but thermally forbidden. Meanwhile, its reverse thermal reaction, such as the pyrolysis of cyclobutane, is a stepwise process with a high energy barrier involving a radical intermediate.

After exposure of **CBDA-1** under sunlight for a month or UV irradiation with a Hanovia medium pressure mercury lamp for two weeks, the sample showed no change in its FT-IR and^1^H NMR spectra. Comparison of the UV-Vis spectra of **CBDA-1** and its starting material *trans*-cinnamic acid showed a clear blue shift with the maximum absorption moving from 270 nm to 208 nm after photoreaction (*see* Supplementary Figure S4). The formation of two *sp* ^3^ carbons between the phenyl ring and carboxylic acid group led to the deconjugation, which is responsible for the shift and sunlight stability of **CBDA-1**.

In addition to thermal and sunlight stability, the cyclobutane ring of **CBDA-1** also shows good chemical stability. No changes were observed after boiling **CBDA-1** in 6 M HCl at 100 °C for 24 hours and its^1^H NMR spectrum confirmed that there was no isomerization or any other changes. As expected, **CBDA-1** reacted with KOH via acid-base neutralization reaction. After boiling **CBDA-1** in 15 M KOH aqueous solution for 24 hours, the mixture was acidized with HCl to pH = 3 and **CBDA-1** was precipitated out and filtered. Its^1^H NMR showed no change.

### Single crystal X-ray diffraction analysis

**Figure 4:**
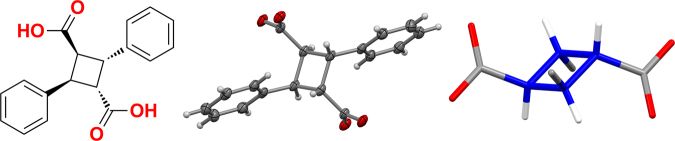
Structure of **CBDA-1**: (left) chemical structure of the building block; (middle) X-ray single crystal structure in Oak Ridge Thermal Ellipsoid Plot (ORTEP) representing at 50% electron density of monomer structure; (right) single crystal structure in stick style with the cyclobutane ring highlighted in blue (two phenyl groups are omitted for clarity).

The single crystal structure of **CBDA-1** obtained in our lab was consistent with the literature report^38^. Cyclobutane rings in the structure adopted two different orientations appearing in an alternating fashion within linear hydrogen bond chains, which were randomly dispersed throughout the crystal matrix, resulting in a disordered structure (see Supplementary Figure S1). This may have occurred because the relatively small cyclobutane ring did not fully fill the empty space generated in the crystal matrix by the hydrogen bond chain and the relatively rigid structure of **CBDA-1**. A straightforward single crystal structure was obtained by preparing a **CBDA-1** salt, *α*-truxillate-dibutylaminium, in the solvent solution ethanol/water/dimethylformamide (1: 1: 1). The short-chain flexible cation, butylaminium, does not destroy the structure of **CBDA-1**, but can be used to fill spaces in the crystal lattice reducing the chance of disorder. As expected, the single crystal of the **CBDA-1** salt shows that the four carbon atoms on the cyclobutane ring are coplanar and have carbon-carbon bond distances of around 1.57 Å (Fig. 4). The two carboxylic groups on opposite sides of the cyclobutane ring have a 180° angle between them and are offset by 1.40 Å, which is a unique characteristic compared to other well-known diacids. The distance between the two carboxylic groups is 4.76 Å. This distance is similar to the distance between two carboxylic groups on furan-based building block 2,5-furandicarboxylic acid as shown in Fig. 1. The spatial orientation and distance of the two carboxylic acid groups makes it suitable for polymer construction. The cyclobutane ring has two exchangeable conformations, planar and puckered, with about 23 degrees difference between them^48^. The limited conformational freedom of the cyclobutane ring is expected to give **CBDA-1** a unique semi-rigid character^27,49,50^.

### Synthesis of poly-*α*-truxillate

**Figure 5:**
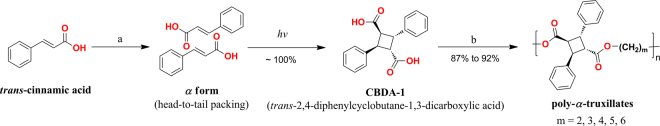
The synthesis of **CBDA-1** and poly-*α*-truxillate. (**a**) The commercially available *trans*-cinnamic acid is in *α-*form (head-to-tail packing). Further crystallization is not necessary. (**b**) Diol, DCC, DMAP, CH_3_CN, r.t. 18 h.

**Figure 6:**
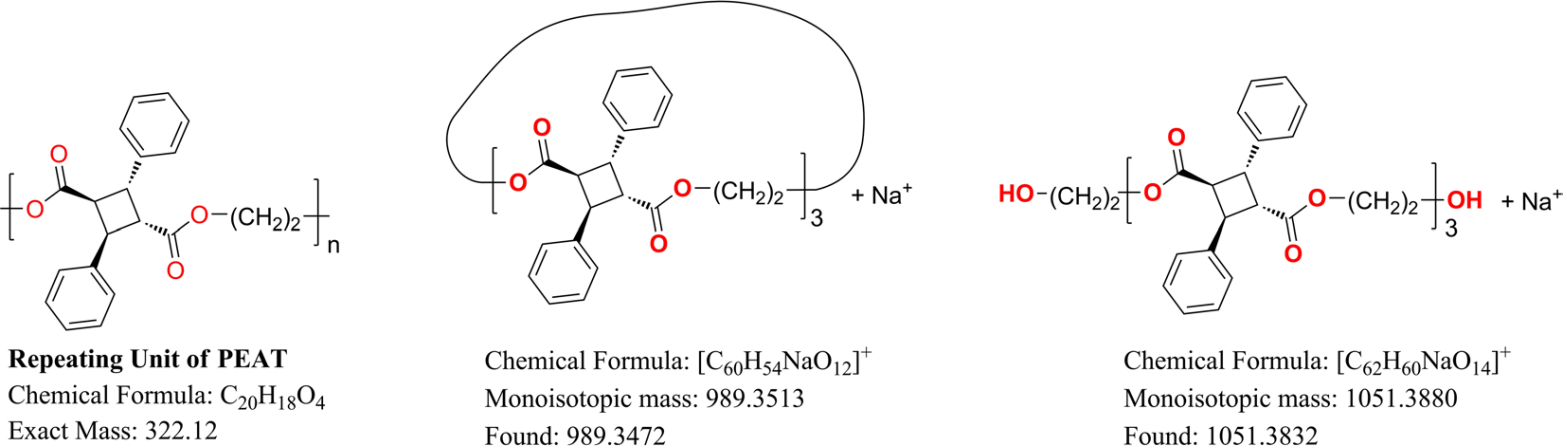
The repeating unit of PEAT (left), cycled PEAT (middle), and linear PEAT (right).

To investigate the possibility of using **CBDA-1** as a building block in materials, it was polymerized with a series of linear diols through a condensation reaction (Fig. 5). Five poly-*α*-truxillates were produced, poly(ethylene-*α*-truxillate) (PEAT), poly(propylene-*α*-truxillate) (PPAT3), poly(1,4-butylene-*α*-truxillate) (PBAT), poly(1,5-pentylene-*α*-truxillate) (PPAT), poly(1,6-hexylene-*α*-truxillate) (PHAT). Out of the five polymers synthesized, only PPAT3 was a liquid at room temperature, so it is not included in the following discussion. Powder X-ray diffraction patterns of these four poly-*α*-truxillates showed that they are semi-crystalline (see Supplementary Figure S12). Two series of peaks were observed in the HRMS spectrum of PEAT (Fig. 6), which was determined by preliminary MALDI-TOF analysis. They have a repeating unit with *m/z* = 322.12 that corresponds with the unit mass of PEAT (C_20_H_18_O_4_ *m/z* = 322.12). One repeating peak of PEAT is ‘*m/z* = 322.12 × n + 22.99 (Na^+^)’ which may indicate that some cycled polyesters exist in the product. For example, ‘*m/z* = 322.12 × 3 + 22.99 = 989.35’. Another repeating peak of PEAT is ‘m/z = 322.12 × n + 62.04 (end-group) + 22.99 (Na^+^)’. This result suggests there are linear polyesters with end-groups HO-(CH_2_)_2_- and –OH. For example, ‘m/z = 322.12 × 3 + 62.04 + 22.99 = 1051.39’. The MS analysis of PEAT revealed both linear and cycled fragments might be present in PEAT. This phenomenon of two different repeating peaks in HRMS spectra was also observed in PBAT, PPAT, and PHAT. Both NMR and HRMS spectra indicated that PEAT was the only compound with a significant number of cycled products. This is probably because it is difficult to form cycled products when the linker molecules contain long and flexible carbon chains. The maximum molecular weights observed in HRMS spectra indicate hexamers. The GPC curves of the poly-*α*-truxillates in THF solution showed a narrow molecular weight distribution with the polydispersity indices (PDI) within the 1.47–1.88 range (see Supplementary Table S2).

### Thermal, chemical, and photochemical property analyses

**Figure 7:**
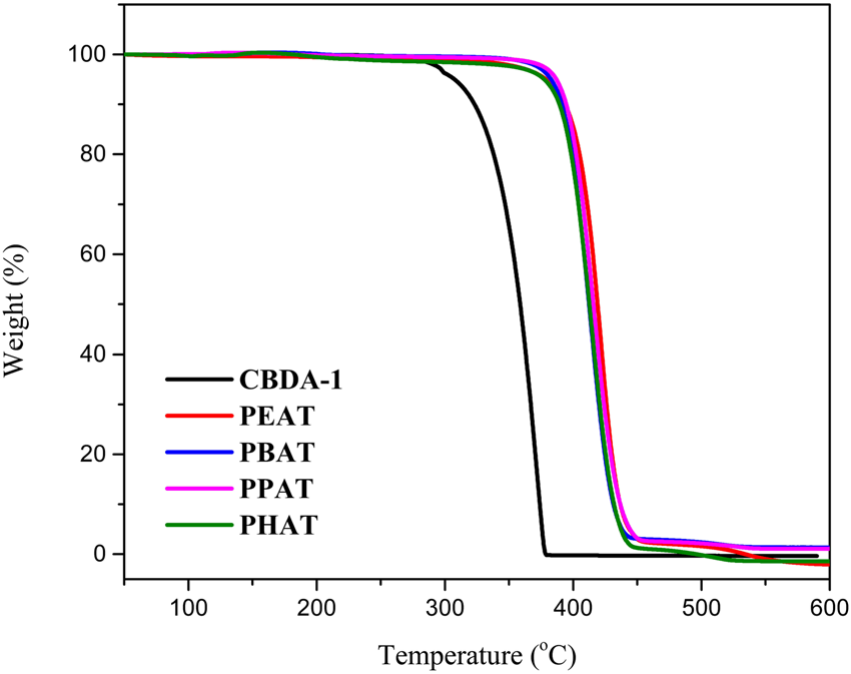
TGA of poly-*α*-truxillates: recorded from 50 °C to 600 °C with a heating rate of 20 °C min^−1^ under N_2_ atmosphere.

Thermogravimetric analysis (TGA, Fig. 7) results indicated the poly-α-truxillates started to decompose around 350 °C, which are comparable to the thermostabilities of terephthalic acid or 2,5-furandicarboxylic acid based polyesters^51^. DSC was used to analyze the glass transition temperature (*T*_g_) of the four polyesters. Supplementary Figure S17 shows a decreasing trend of the poly-α-truxillates’ *T*_g_s with increasing diol carbon chain length. The *T*_g_ of PEAT is 81 °C whereas the *T*_g_ of PPAT is 64 °C. This trend in the *T*_g_s may be attributed to the increased flexibility of longer carbon chains, which make it easier for the polyester to rotate or twist. After the first heating and cooling process, the DSC curve showed an obvious decreasing trend of *T*_g_s in the second heating process. This phenomenon may be due to the annealing effect of the first heating process. After heating polyesters to 250 °C, the polyesters will be scattered and mixed equally, which may lead to the decreasing *T*_g_s. The poly-α-truxillates showed excellent chemical stability. When treated with common solvents such as diethyl ether, hexane, chloroform, acetonitrile, acetone, DCM, THF, EtOH, EtOAc, and DMSO, the poly-α-truxillates showed no degradation. Hydrolysis was not observed after 24 hours refluxing the poly-α-truxillates in 5 M aqueous NaOH or 6 M HCl. Photochemical stability tests were carried out under ultraviolet irradiation using a Hanovia medium pressure mercury lamp and were monitored by FT-IR and NMR. The preliminary results showed slight degradation of the poly-α-truxillates after continuous ultraviolet irradiation for one week, which was similar to PET^52^.

## Discussion

Both starting materials of the polymers, the diols and cinnamic acid, can be obtained from biomass^43^. While diols have been widely used in making plastics, cinnamic acid has found its applications in flavors, perfumes, synthetic indigo, and certain pharmaceuticals^39^. It has recently been adequately derived from a side product of biofuel manufacture and from other renewable sources including glucose via engineered *E. coli* ^53–55^. Biomass-derived cinnamic acid has also been reported to be used for styrene synthesis through a decarboxylation reaction to produce environmentally friendly polystyrene^56^. Meanwhile, the photoreaction to synthesize **CBDA-1** was able to be carried out under sunlight. Thus, **CBDA-1** could be produced as a greener diacid to replace or partially replace commodity petrochemicals such as terephthalic acid in the future.

## Conclusion

A promising building block **CBDA-1** was introduced to materials synthesis in this study. The simple preparation of **CBDA-1** has been demonstrated and discussed. Our study has shown that using **CBDA-1** in the construction of materials is beneficial not only because it can be produced from bio-based starting materials, but also because it has thermal, sunlight, and chemical stability. Moreover, the four-membered carbon ring structure of **CBDA-1** offers a unique semi-rigid property for materials. These features of **CBDA-1** allow it to be used directly in making new polymers or be added into known polymer recipes in certain ratio to modify their physical properties such as transparency and glass transition temperature (*T*_g_)^39,57^. The successful synthesis of a new family of polyesters presented in this work confirmed that **CBDA-1** is a useful building block for polymers. Initial thermal, chemical, and photochemical analyses revealed stabilities of the newly synthesized cyclobutane-containing polyesters which are comparable to those of PET. As novel building blocks, **CBDA-1** and its congeners will provide great opportunities in producing a variety of materials (e.g., polyesters, polyamides, polycyclobutanes, copolymers, and coordination polymers) with new properties and applications^58–60^.

## Methods

### Solid state photodimerization

Commercially available *trans*-cinnamic acid (2 g) was scattered on a 10″ × 10″ glass plate. The plate was put outside in sunlight or in a photoreactor with UV lamp. The process of photodimerization was monitored by FT-IR which showed completion after 10 h under sunlight and 8 h under a mercury lamp, respectively. About 1.96 g of **CBDA-1** (*trans*-2,4-diphenyl-1,3-cyclobutanedicarboxylic acid or *α*-truxillic acid) was obtained.

### Single crystal growth and characterization

*trans*-Cinnamic acid: To achieve high quality single crystals for X-ray data collection, 60 mg of *trans*-cinnamic acid was added to a mixture of ethyl acetate and acetonitrile (1:1, 20 mL). The mixture was allowed to stand in the fume hood until single crystals were formed. **CBDA-1**: 10 mg of *α*-truxillic acid was dissolved in 20 mL of ethanol. The mixture was sonicated for 30 min. The obtained clear solution was allowed to stand at room temperature without cover for three days or until the crystals were formed. **CBDA-1 salt** (*α*-truxillate-dibutylaminium): 20 mg of *α*-truxillic acid was dissolved in DMF (5 mL) in a 20 mL vial. A mixture of ethanol (5 mL), water (5 mL) and *n*-butylamine (0.5 mL) was then added to this solution. The mixture was heated to 70 °C and stirred for 10 mins. Crystals were obtained in around three days.

### General procedure for poly-*α*-truxillate synthesis. CBDA-1

Ethylene glycol, *N*,*N*′-dicyclohexyl-carbodiimide (DCC) and 4-dimethylaminopyridine (DMAP) were added to a solvent of acetonitrile (CH_3_CN, 20 mL). The mixture was stirred at room temperature for 20 h and mixed with 20 mL of chloroform. The mixture was filtered via column chromatography to give product poly(ethylene-*α*-truxillate) (PEAT) as a white solid.

### Data availability

The data supporting the findings of this study are available within the article and its Supplementary Information files. All data are available from the corresponding author upon reasonable request.

The crystallographic data for *trans*-cinnamic acid, **CBDA-1**, and **CBDA-1** dibutylaminium salt are available in Cambridge Crystallographic Data Centre (CCDC# 986274, 1547787, and 1547788).

## Electronic supplementary material

Supporting Information
