# Supplementary material for: Cyclobutane-1,3-Diacid (CBDA): A Semi-Rigid Building Block Prepared by [2+2] Photocyclization for Polymeric Materials
Source: Sci Rep. 2017 Oct 20;7:13704. doi: 10.1038/s41598-017-13983-z (PMC5651925; doi:10.1038/s41598-017-13983-z)
Supplement: Supplementary file 1 — Supporting Information [file 41598_2017_13983_MOESM1_ESM.pdf]

Cyclobutane-1,3-Diacid (CBDA): A Semi-Rigid Building Block Prepared by [2+2] Photocyclization for Polymeric Materials

Zhihan Wang,[†] Benjamin Miller,[†] Micah Mabin,[†] Rahul Shahni,[†] Zijun D. Wang,[†] Angel Ugrinov,[‡] and Qianli Rick Chu*[,†]

[†]*Department of Chemistry, University of North Dakota, Grand Forks, ND 58202, USA*
[‡]*Department of Chemistry and Biochemistry, North Dakota State University, Fargo, ND 58102, USA*

Table of Contents

1. Chemicals and Instruments 2
2. Crystal Data and Conformations of Cyclobutane Ring 3
   2.1. X-ray Single Crystal Structures of *trans*-Cinnamic Acid, **CBDA-1** Dibutylamine Salt, and **CBDA-1**. 3
   2.2. **CBDA-1** and its Five Possible Stereoisomers 4
   2.3. Crystal Data of the Cinnamic Acid, α-Truxillic Acid and its Salt 5
3. Spectra, Data and Images 6
   3.1. Powder X-ray Diffraction of *trans*-Cinnamic Acid 6
   3.2. UV-Vis Spectra of *trans*-Cinnamic Acid, **CBDA-1**, and Poly-α-Truxillates 7
   3.3. NMR Spectra 8
   3.4. FT-IR of α-Truxillic Acid and Poly-α-Truxillate 23
   3.5. Powder X-ray Diffraction of Poly-α-Truxillate 26
   3.6. MS Spectra 28
   3.7. DSC 32
   3.8. TGA 34
   3.9. PDI .... .34

1. Chemicals and Instruments

All chemicals were purchased from Alfa Aesar, Sigma-Aldrich, or Acros, and used without further purification. The light sources used for the photopolymerization were sunlight or a Hanovia medium pressure mercury lamp (PC 451050, 450 W). The solution phase nuclear magnetic resonance spectra (NMR) were recorded with Bruker AVANCE (^{1}H: 500 MHz, ^{13}C: 125 MHz). Single crystal X-ray data were recorded on Bruker Apex or Bruker Kappa Apex II Duo X-Ray Diffractometer with Mo $K\alpha$ (λ = 0.71073 Å) or Cu $K\alpha$ (λ = 1.54178 Å). Infrared spectroscopy (IR) was recorded on Thermo Scientific Nicolet iS5 FT-IR spectrometer. Melting points were measured on a MEL-TEMP device without correction. The mass spectrometric analyses were performed using a high-resolution time of flight G1969A with electrospray (atmospheric pressure chemical) ionization (Agilent, Santa Clara, CA, USA) and reported as m/z (relative intensity). MALDI was performed on the instrument of Waters SYNAPT G2Si with MALDI source. Differential scanning calorimetry (DSC) was recorded with Perkin Elmer Jade DSC with a ramping rate of 10 °C/min under nitrogen protection. Heat flow was recorded from both the first and second heating cooling curve. Thermogravimetric analysis (TGA) was performed with TA instrument SDT Q600 at a ramping rate 20 °C/min under nitrogen atmosphere. Polydispersity indices (PDI) of polymers were obtained by gel permeation chromatography (GPC) equipped with Waters 1515 Isocratic HPLC pump, Styragel HR 4 column, and 2414 refractive index detector with THF as eluent. Monodisperse polystyrene and poly(methyl methacrylate) samples were employed to construct the calibration curve. X-ray Powder Diffraction (XRD) was performed on a X'PERT-PRO X-ray diffractometer (PANalytical, Netherlands) equipped with a 3 KW copper tube X-ray generator of λ = 0.1541 nm under 40 mA and 45 KV. Spectra were collected at room temperature in a 2θ range of 3~35° at a scanning rate of 3°/min.

2. Crystal Data and Conformations of Cyclobutane Ring

2.1. X-ray Single Crystal Structures of *trans*-Cinnamic Acid, CBDA-1 Dibutylamine Salt, and CBDA-1.

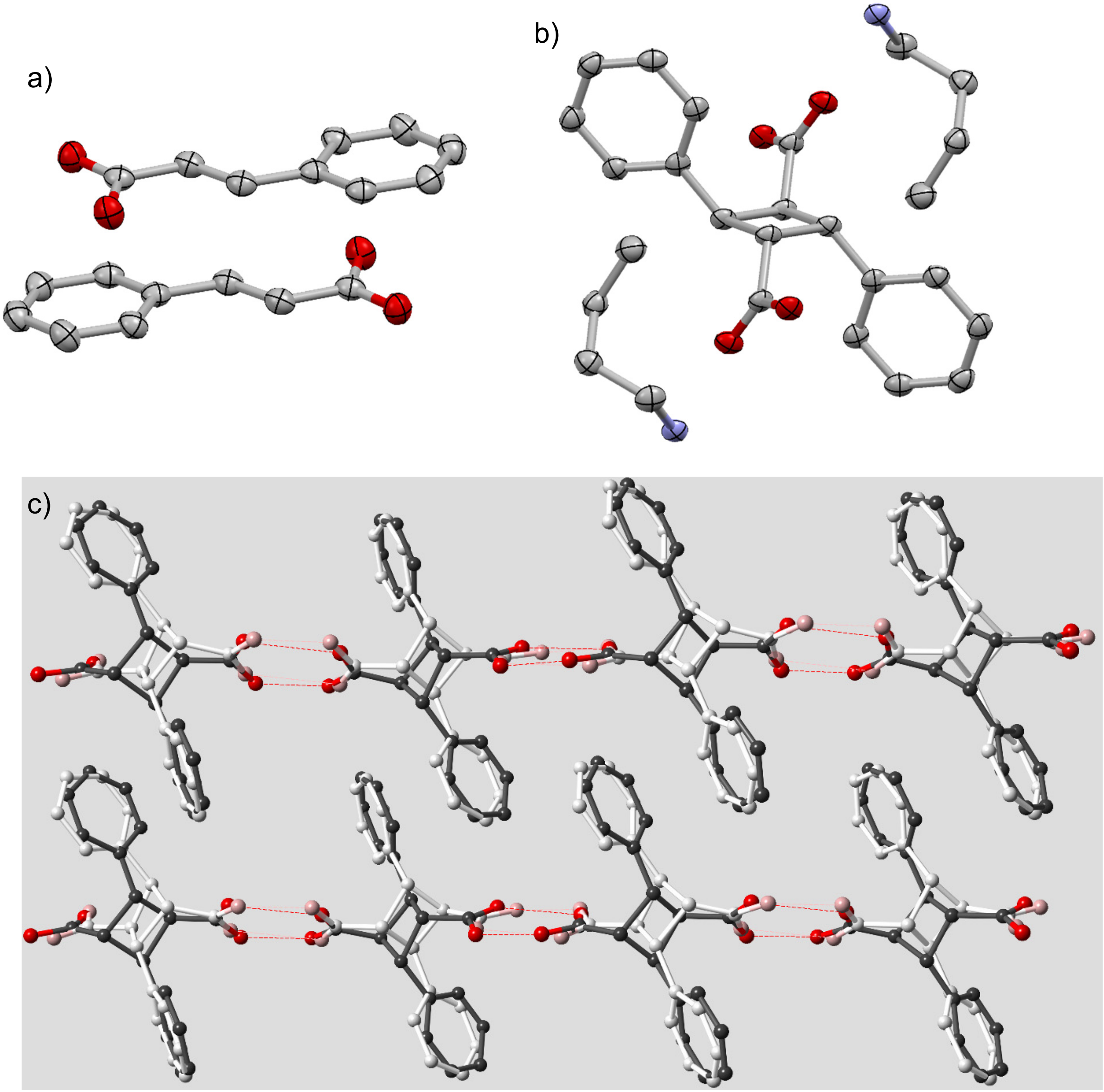

Figure S1. ORTEP representation at 50% electron density: (a) Crystal structure of the *trans*-cinnamic acid; (b) Crystal structure of the **CBDA-1** dibutylamine salt (α-truxillate dibutylaminium); (c) The packing the **CBDA-1** in the its pure crystal form with disorder (The hydrogen bonds are shown in red and pink).

2.2. CBDA-1 and its Five Possible Stereoisomers

Truxillic acid:

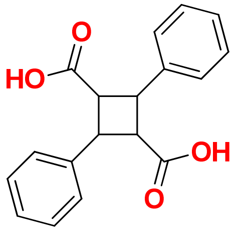

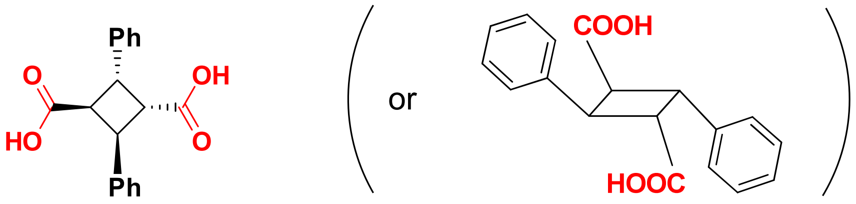

CBDA-1 (*α-truxillic acid*)

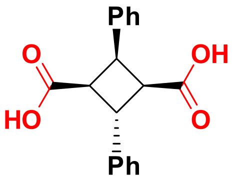

γ-truxillic acid

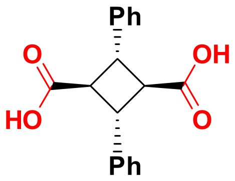

ε-truxillic acid

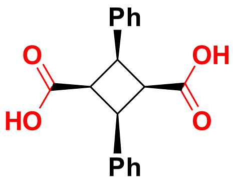

peri-truxillic acid

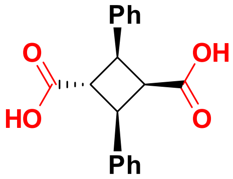

epi-truxillic acid

Figure S2. Truxillic acid: Chemical structures of **CBDA-1** (*α*-truxillic acid, prepared from *trans*-cinnamic acid), and the other four possible stereoisomers of truxillic acid.

2.3. Crystal Data of the Cinnamic Acid, α-Truxillic Acid and its Salt

Table S1. Crystal data

Crystals	*trans*-Cinnamic acid	CBDA-1[a] (α-Truxillic acid)	CBDA-1 Salt (α-Truxillate-dibutylaminium)
**CCDC #**	1547787	986274	1547788
**Formula**	C9H8O2	C18H16O4	C26H38 N2O4
**FW**	148.15	294.29	442.58
**Cryst. Size [mm]**	0.22, 0.17, 0.06	0.22, 0.05, 0.02	0.28, 0.13, 0.07
**Space Group, Z**	P21/n	C2/c	P -1
**a (Å)**	5.5531(4)	15.7822(6)	6.1667(5)
**b (Å)**	17.5178(13)	5.6013(2)	9.3491(6)
**c (Å)**	7.7056(6)	16.2750(5)	10.8378(9)
***α* (°)**	90	90	98.608(5)
**β (°)**	96.267(6)	99.255(3)	91.191(7)
**γ (°)**	90	90	96.935(5)
**V (Å^3)**	745.11(10)	1420.00(9)	612.79(8)
**Temp. (K)**	103(2)	100(2)	100(2)
**ρcalc [g/cm^3]**	1.321	1.377	1.199
**μ [mm^{-1}]**	0.764	0.801	0.640
**Radiation Type**	Cu	Cu	Cu
**F(000)**	312.0	616.0	240.0
**No of measured refl.**	3308	4256	7270
**No of independent refl.**	1237	1231	2108
**No of refl. (I ≥ 2σ)**	1016	1091	1751
**R1/wR2 (I ≥ 2σ) [%]**	4.07/11.12	5.02/13.45	8.40/22.84
**R1/wR2 (all data) [%]**	4.95/11.75	5.57/13.82	9.37/24.31

a) Data from our recent publication: Wang, Z.; Randazzo, K.; Hou, X.; Simpson, J.; Struppe, J.; Ugrinov, A.; Kastern, B.; Wysocki, E.; Chu, Q. R. **2015**, *48(9)*, 2894-2900.

3. Spectra, Data and Images

3.1. Powder X-ray Diffraction of *trans*-Cinnamic Acid

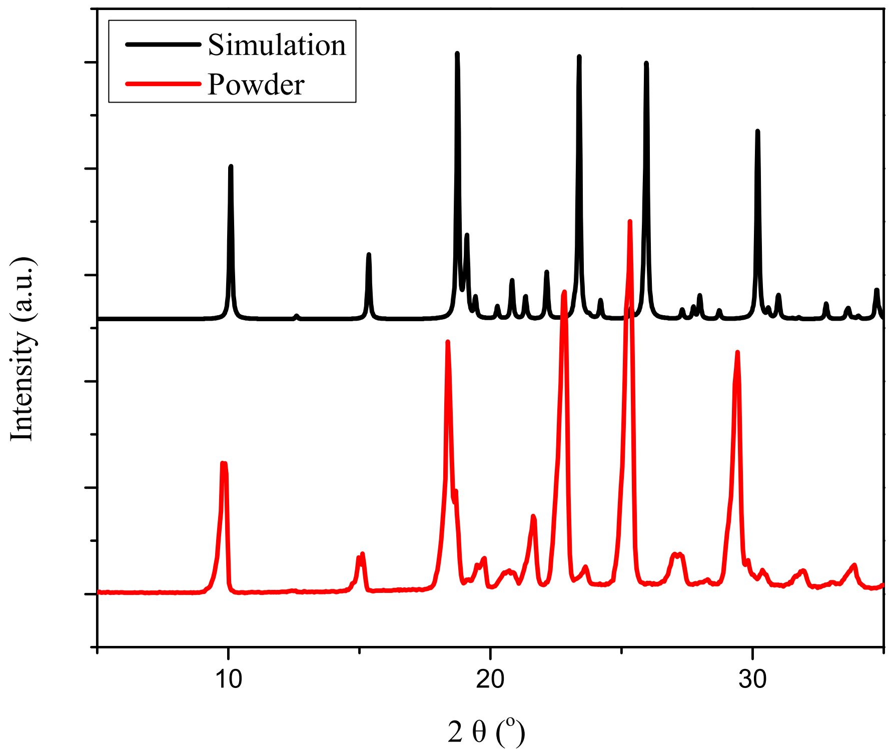

Figure S3. Comparison of the calculated Powder X-ray Diffraction (PXRD) pattern based on single crystal structure of the α form *trans*-cinnamic acid (black line) and the one of the commercially available *trans*-cinnamic acid powder (red line).

3.2. UV-Vis Spectra of *trans*-Cinnamic Acid, CBDA-1, and Poly-α-Truxillates

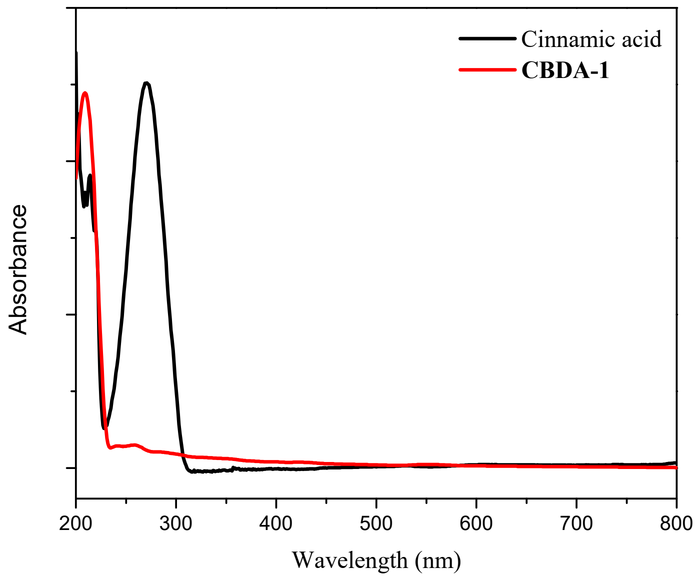

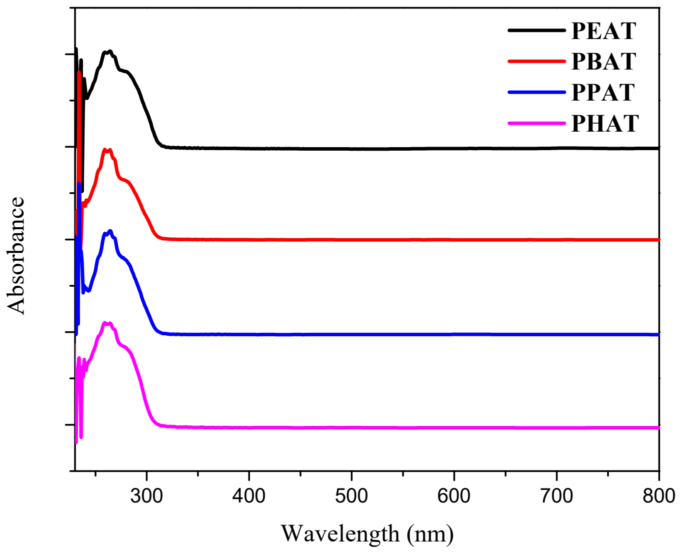

Figure S4. UV-Vis spectra of *trans*-cinnamic acid and **CBDA-1** in ethanol (up) and poly-α-truxillates in chloroform (down).

3.3. NMR Spectra

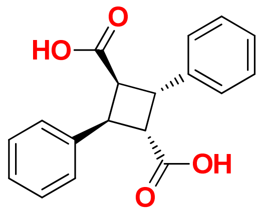

CBDA-1 (α-truxillic acid)

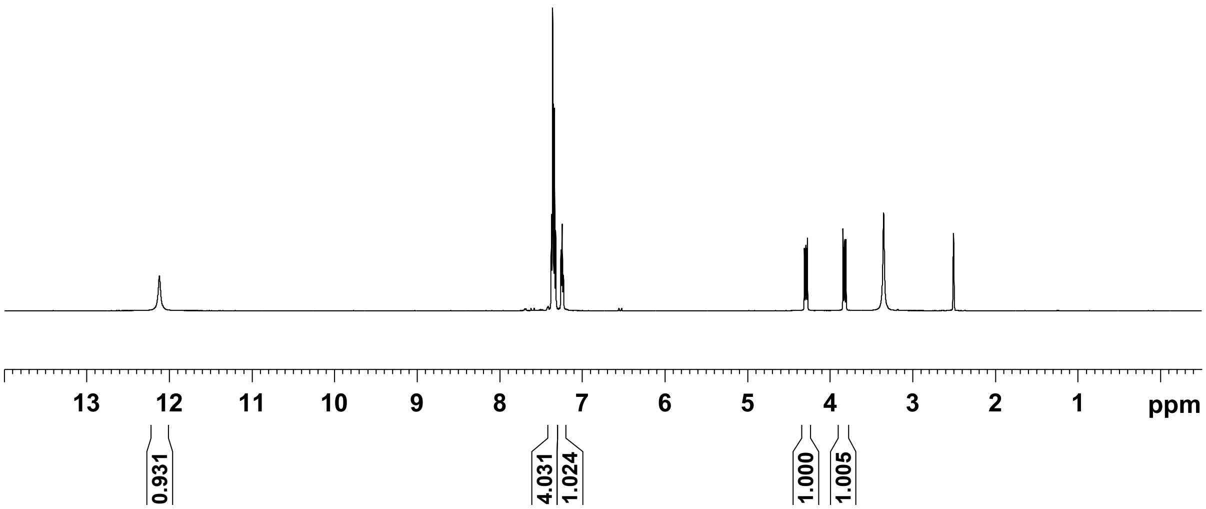

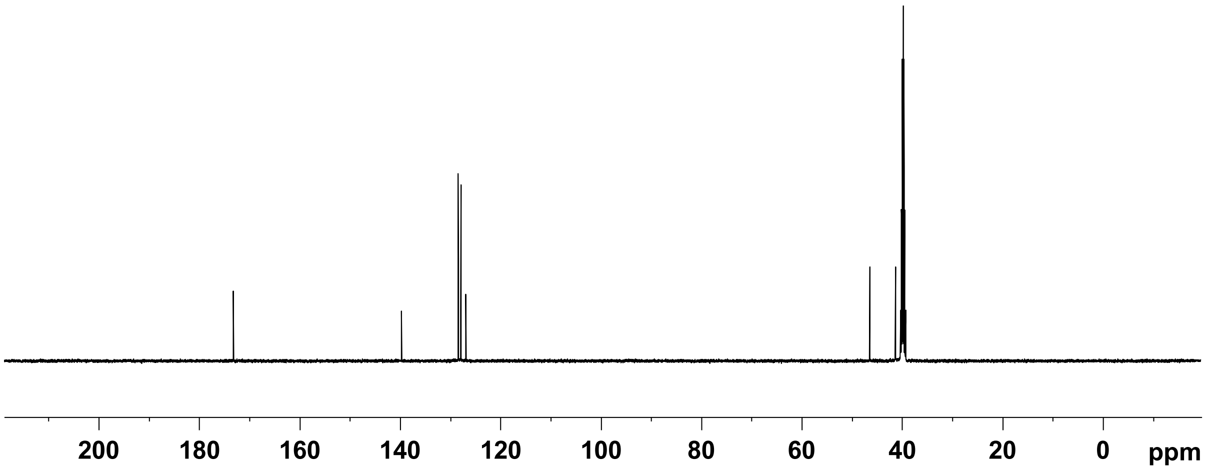

Figure S5. ^{1}H (a) and ^{13}C (b) NMR spectura of **CBDA-1** (α-truxillic acid) in DMSO-d_6 at room temperature.

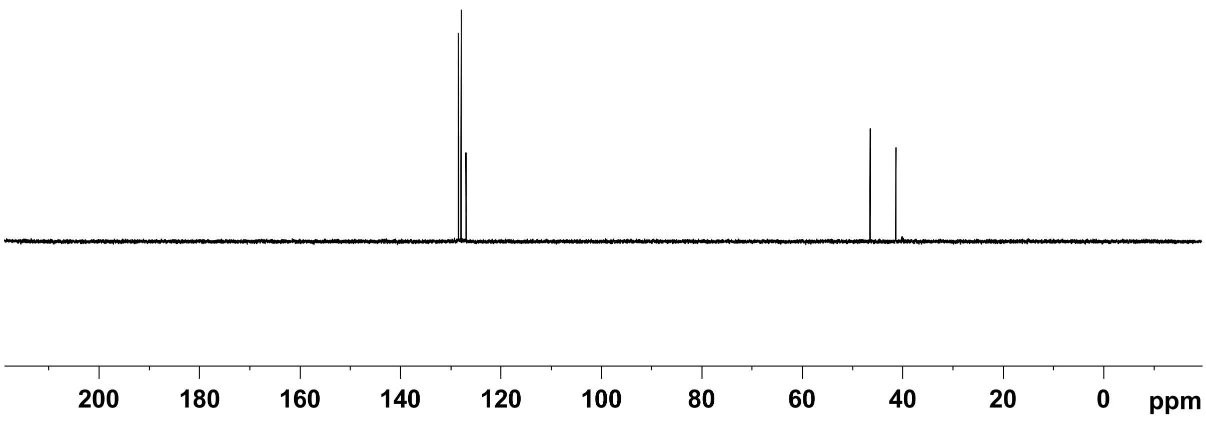

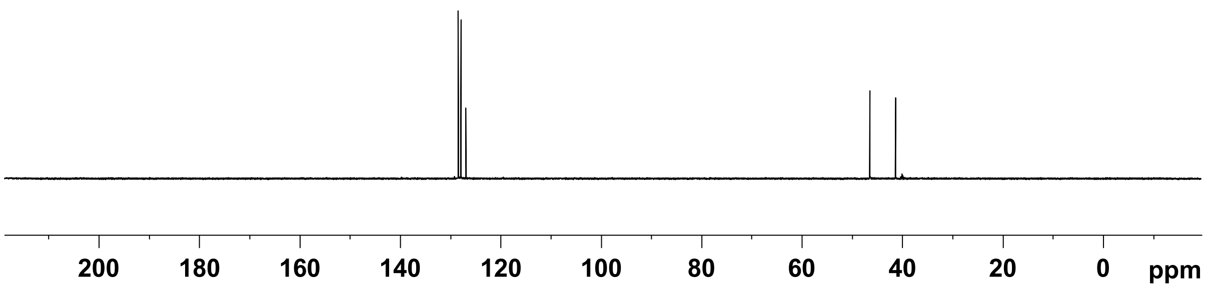

Figure S5 Continue. DEPT 135 (c) and DEPT 90 (d) ^{13}C NMR spectura of **CBDA-1** in DMSO-d_6 at room temperature.

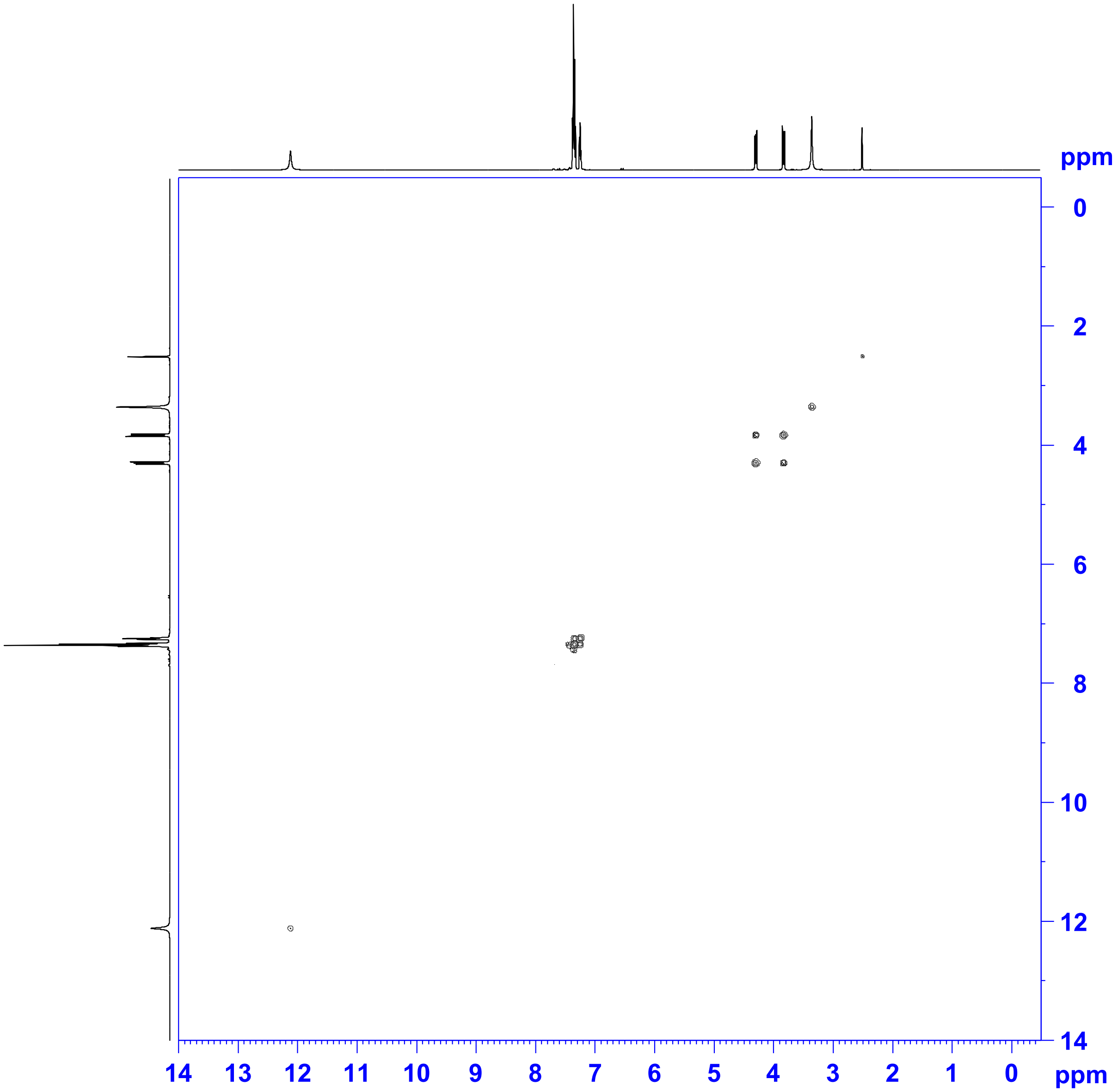

Figure S5 Continue. COSY NMR spectrum of **CBDA-1** in DMSO-d_6 at room temperature.

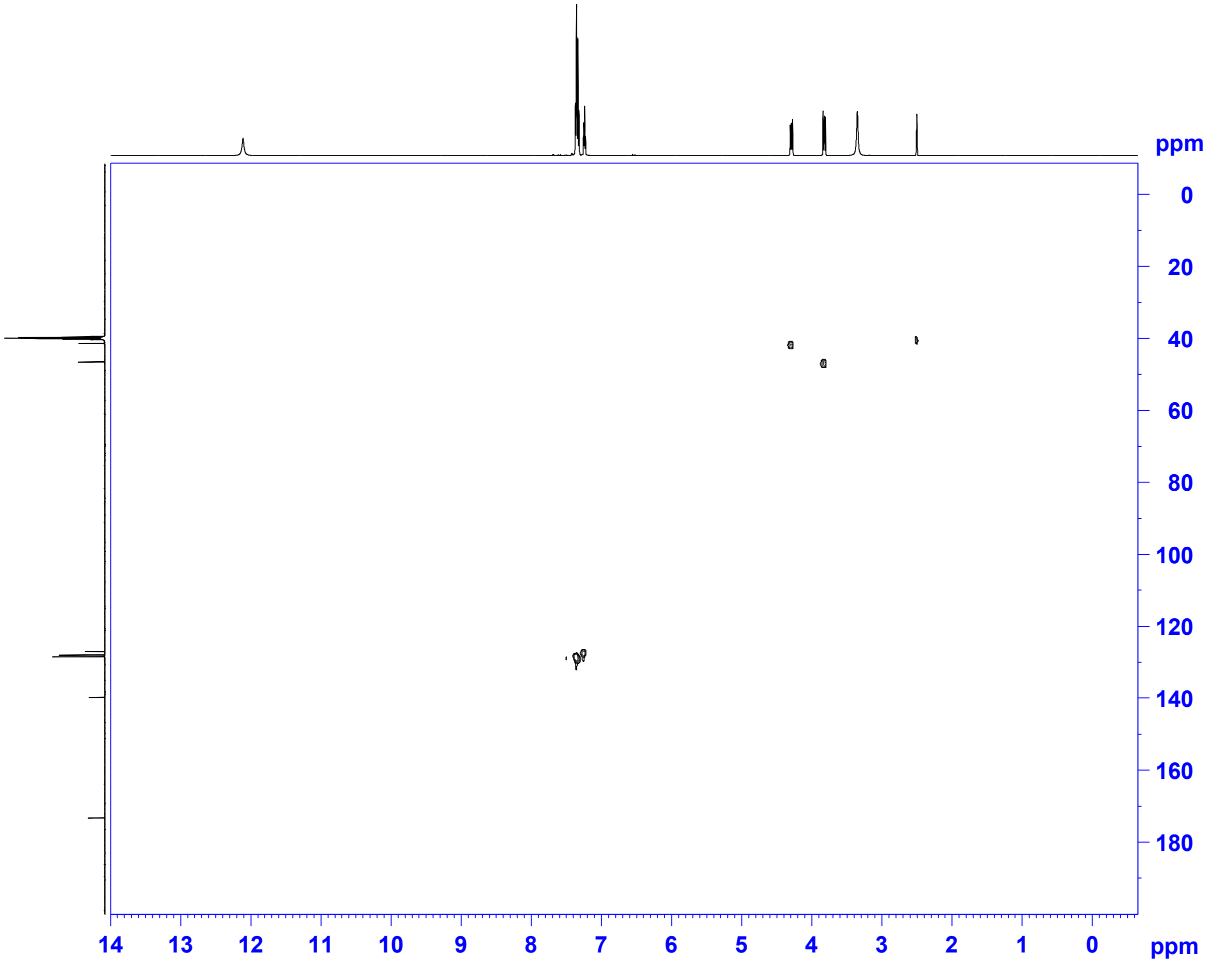

Figure S5 Continue. HSQC NMR spectrum of **CBDA-1** in DMSO-d_6 at room temperature.

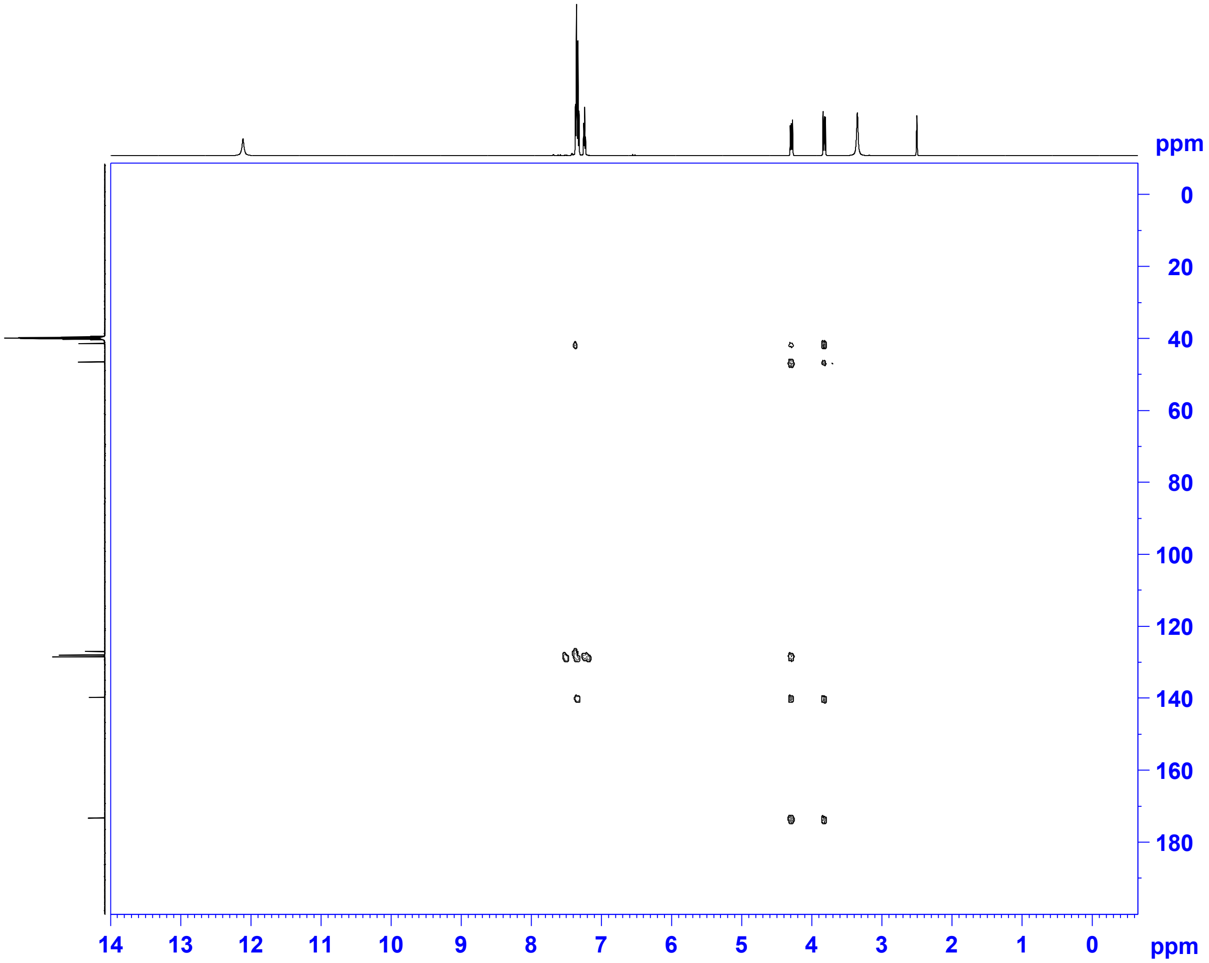

Figure S5 Continue. HMBC NMR spectrum of **CBDA-1** in DMSO-d_6 at room temperature.

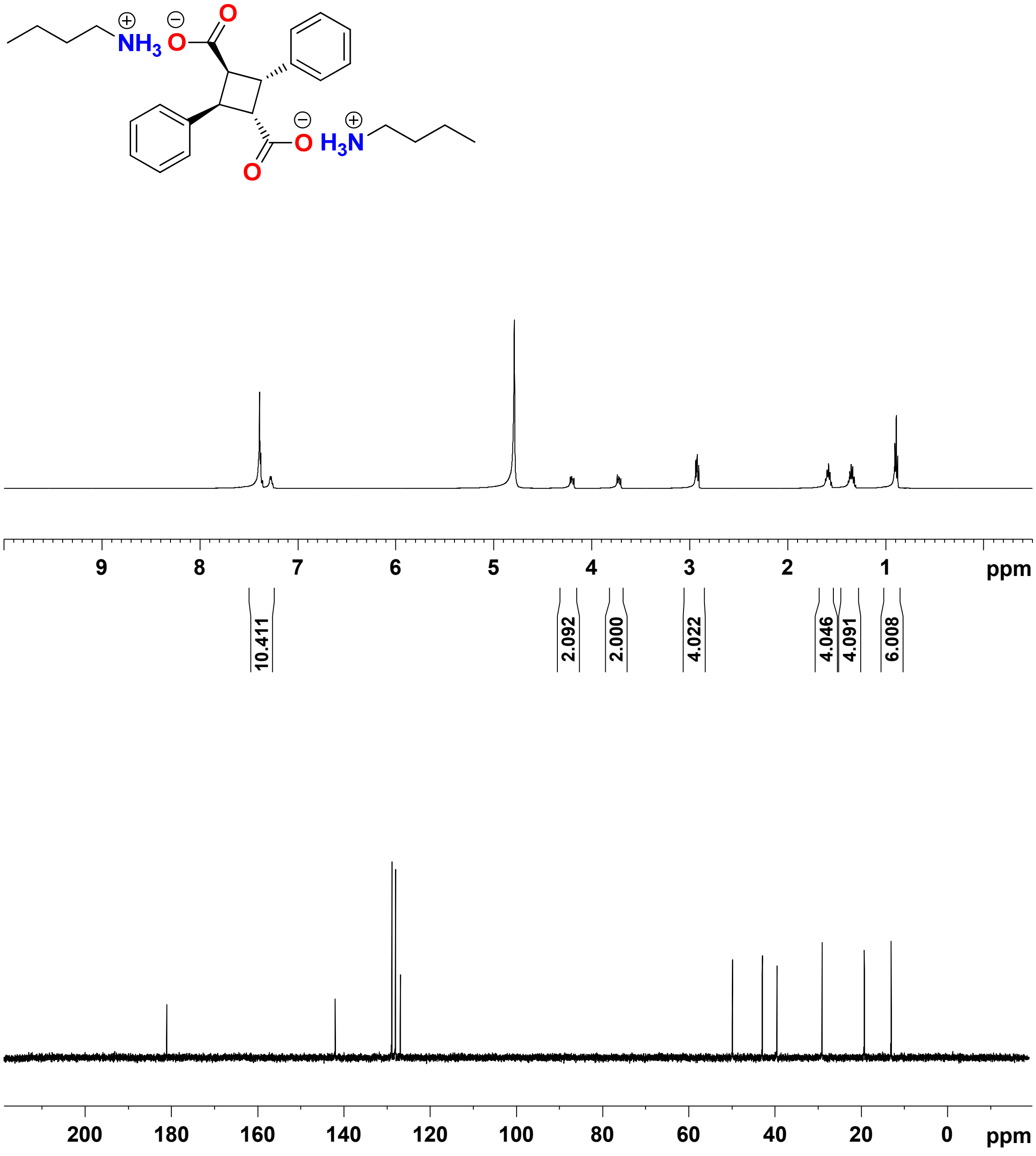

Figure S6. 1H and 13C NMR spectra of **CBDA-1** dibutylaminium salt in D_2O at room temperature.

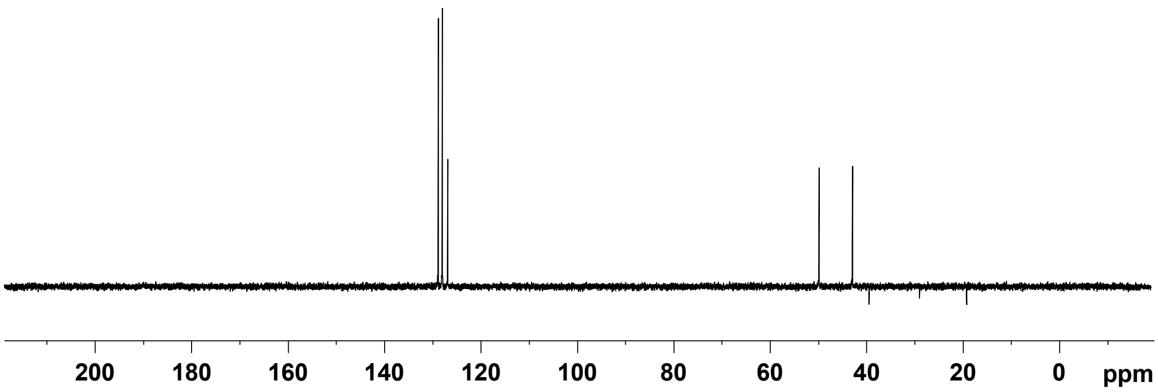

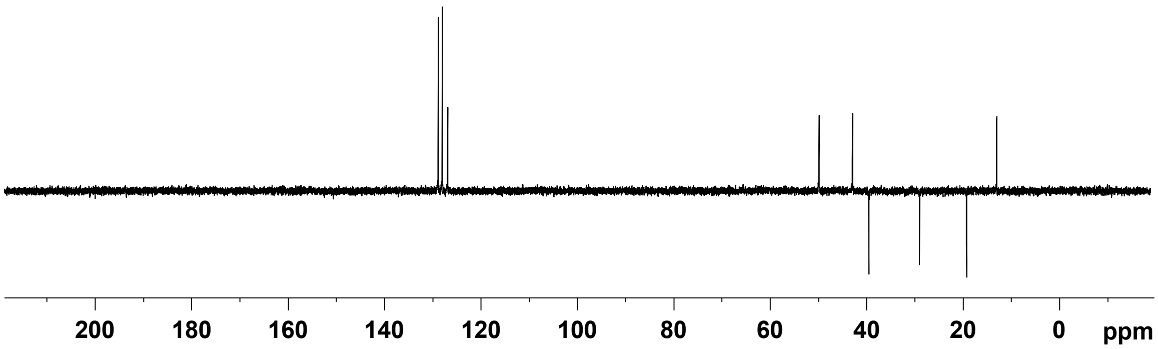

Figure S6 continue. DEPT 90 and DEPT 135 NMR spectra of **CBDA-1** dibutylaminium in D_2O at room temperature.

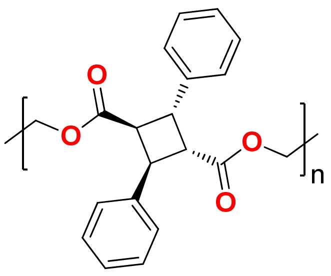

PEAT

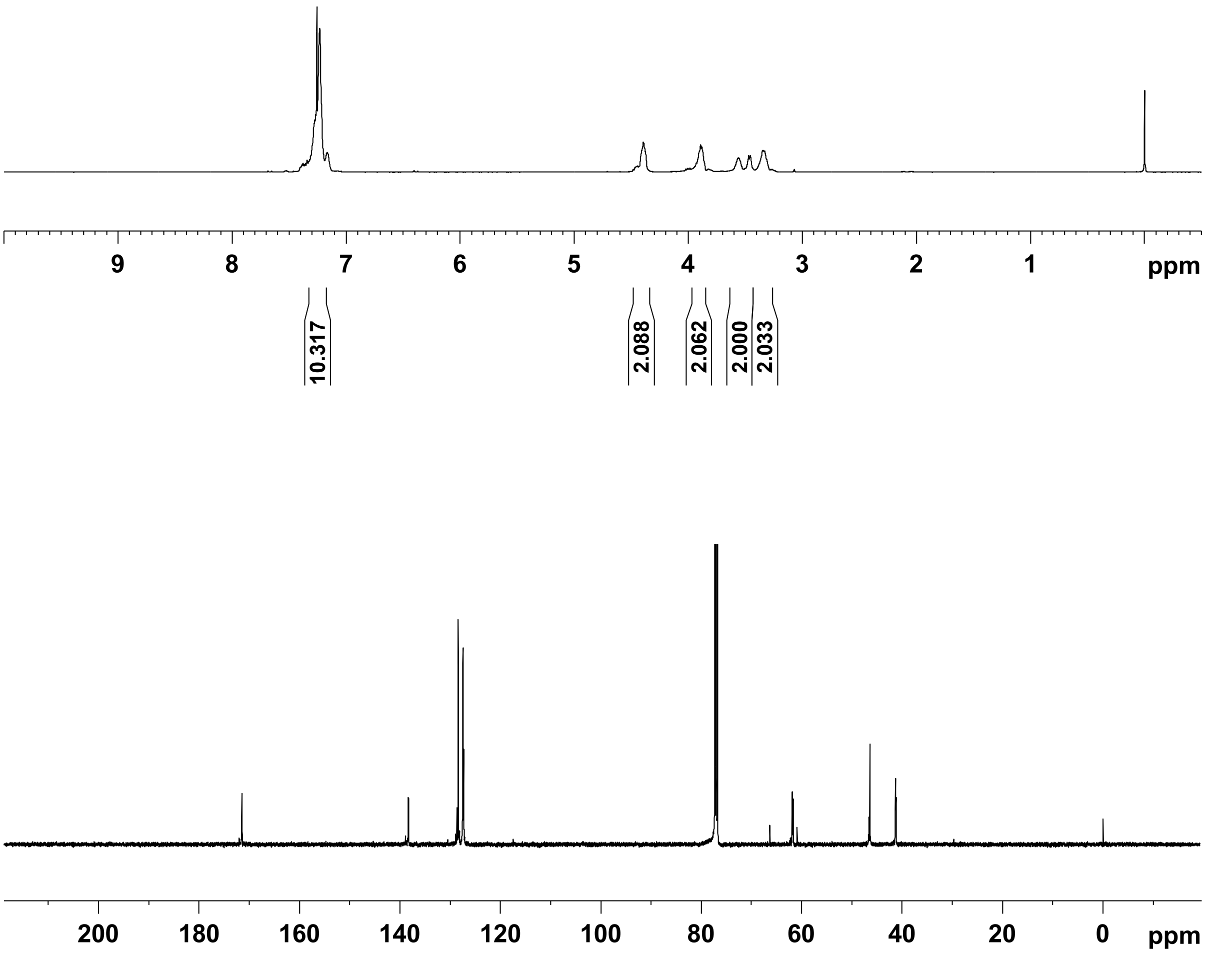

Figure S7. ^{1}H (a) and ^{13}C (b) NMR spectra of PEAT in $CDCl_3$ at room temperature.

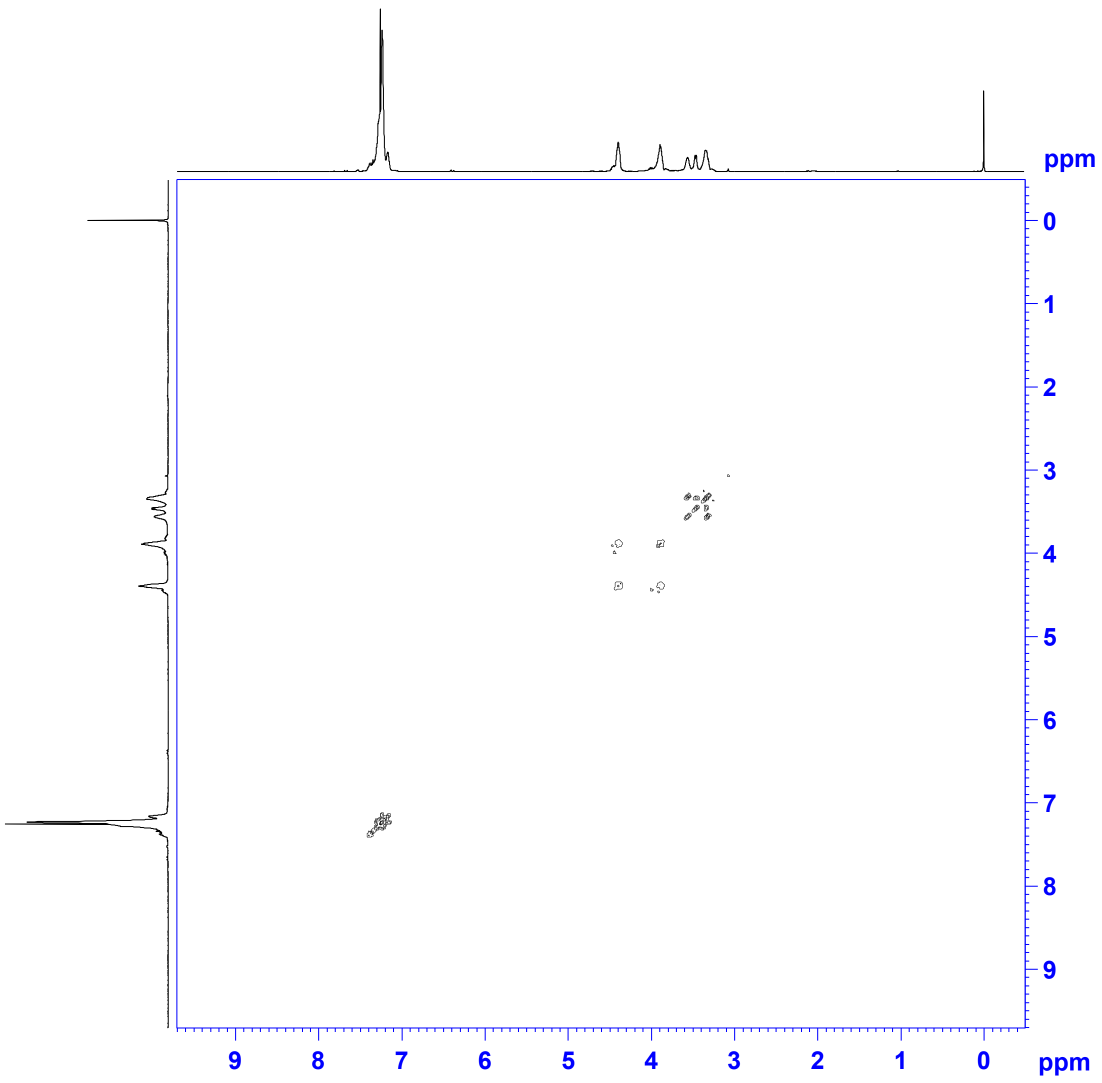

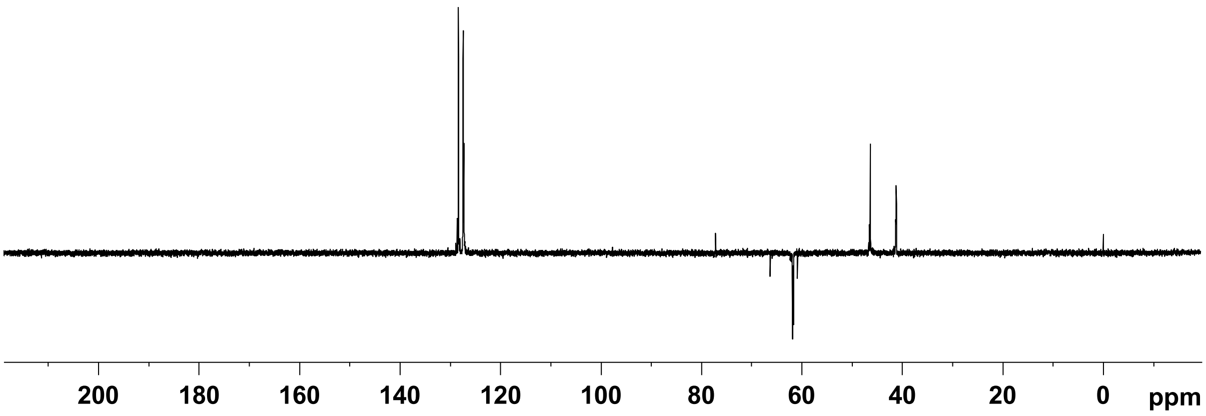

Figure S7 continue. ^{1}H COSY (c) and ^{13}C DEPT 135 (d) NMR spectra of PEAT in $CDCl_3$ at room temperature.

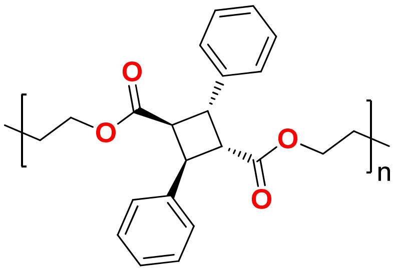

PBAT

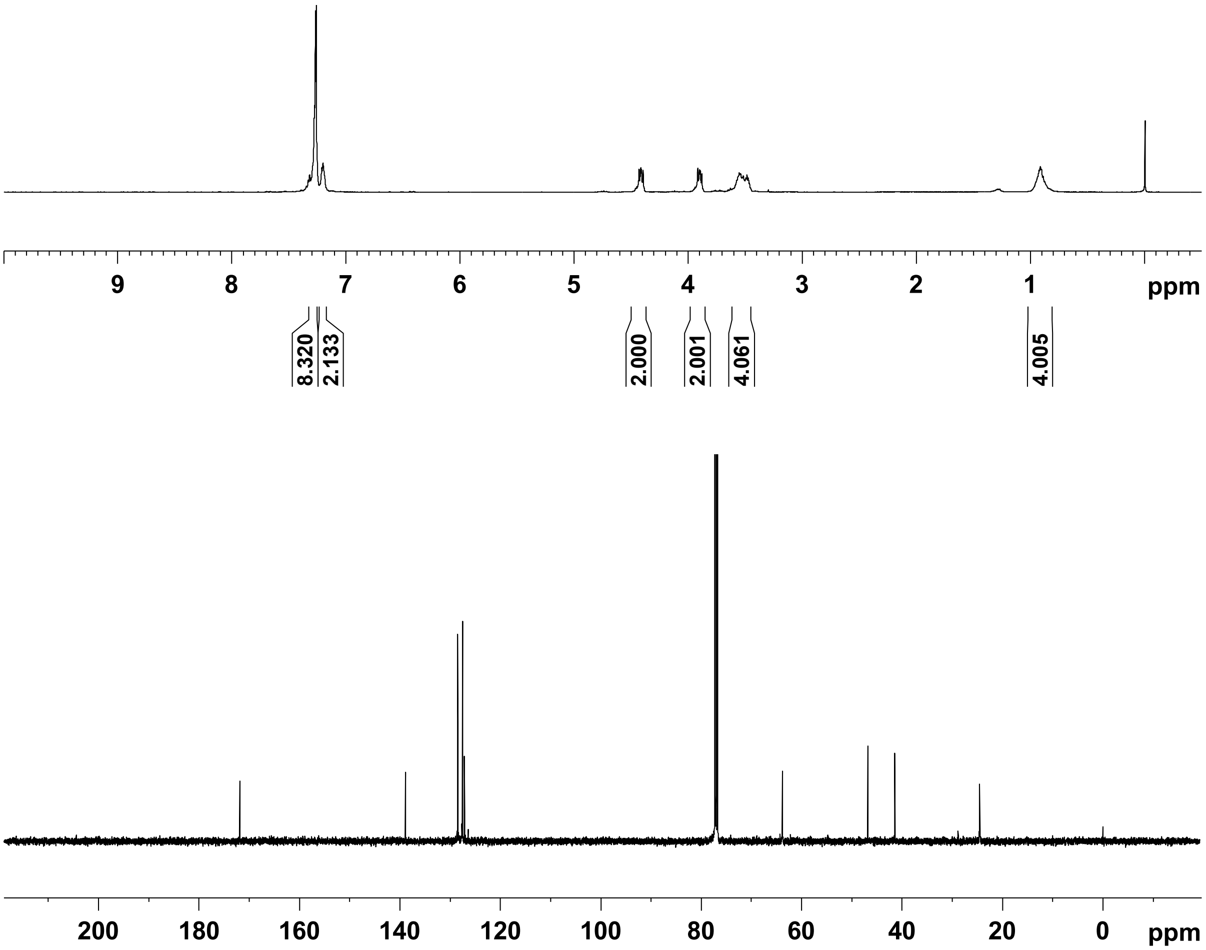

Figure S8. ^{1}H (a) and ^{13}C (b) NMR spectra of PBAT in $CDCl_3$ at room temperature.

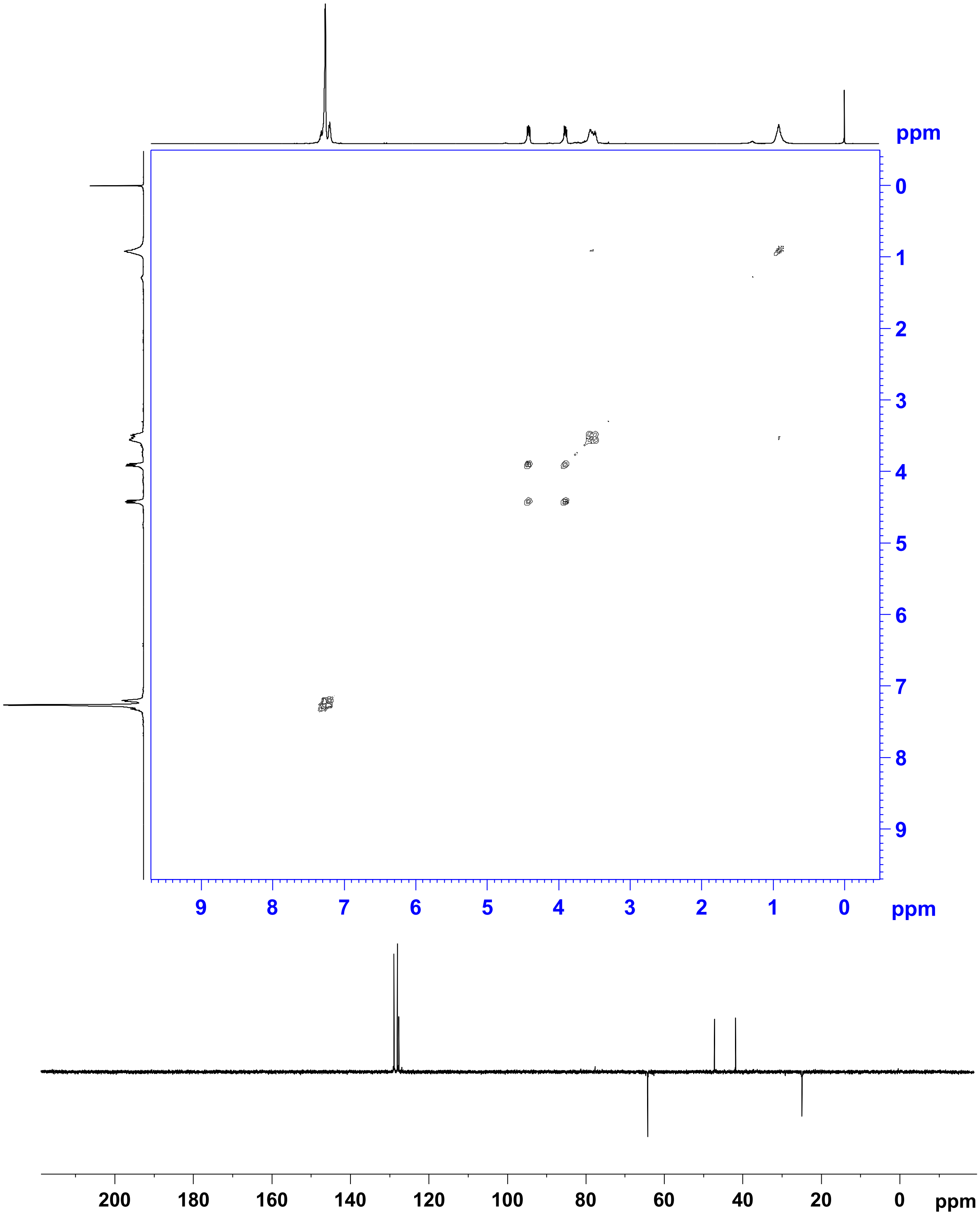

Figure S8 continue. ^{1}H COSY (c) and ^{13}C DEPT 135 (d) NMR spectra of PBAT in $CDCl_3$ at room temperature.

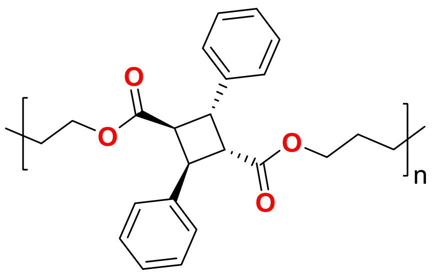

PPAT

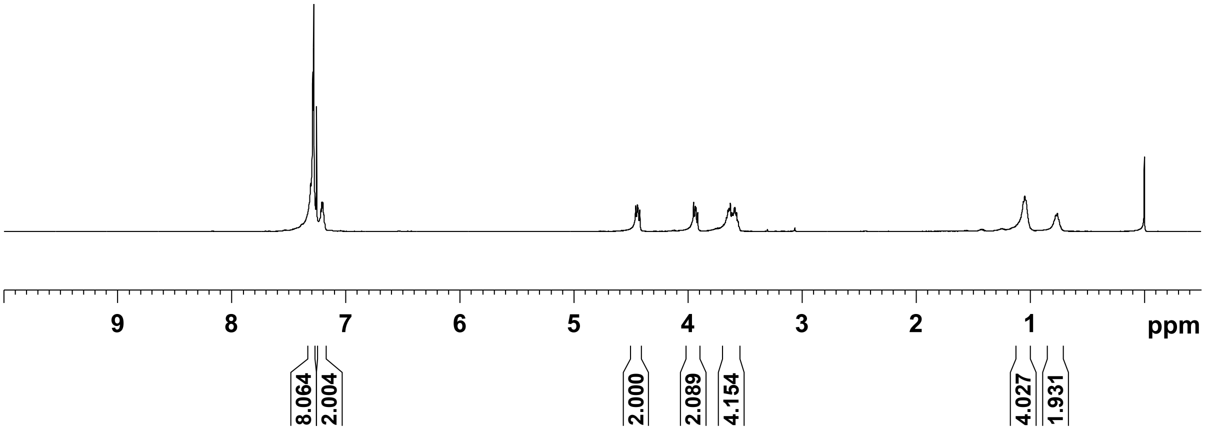

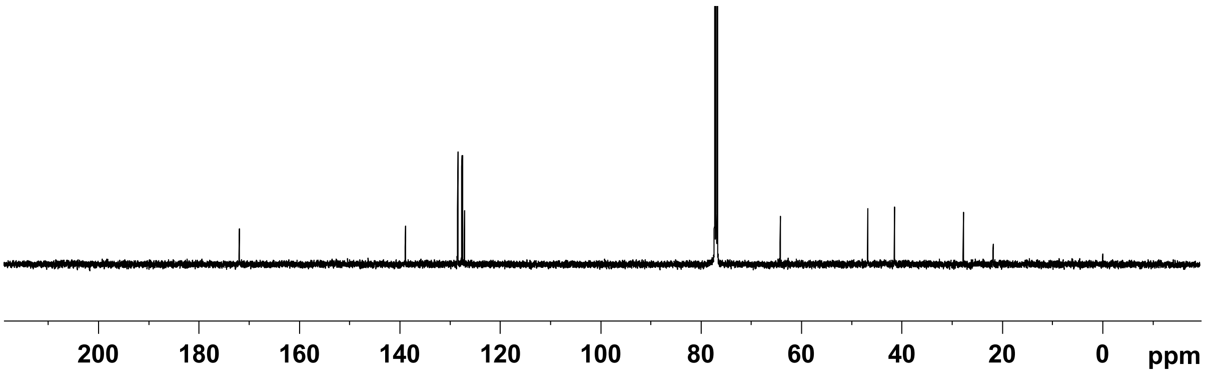

Figure S9. ^{1}H (a) and ^{13}C (b) NMR spectra of PPAT in $CDCl_3$ at room temperature.

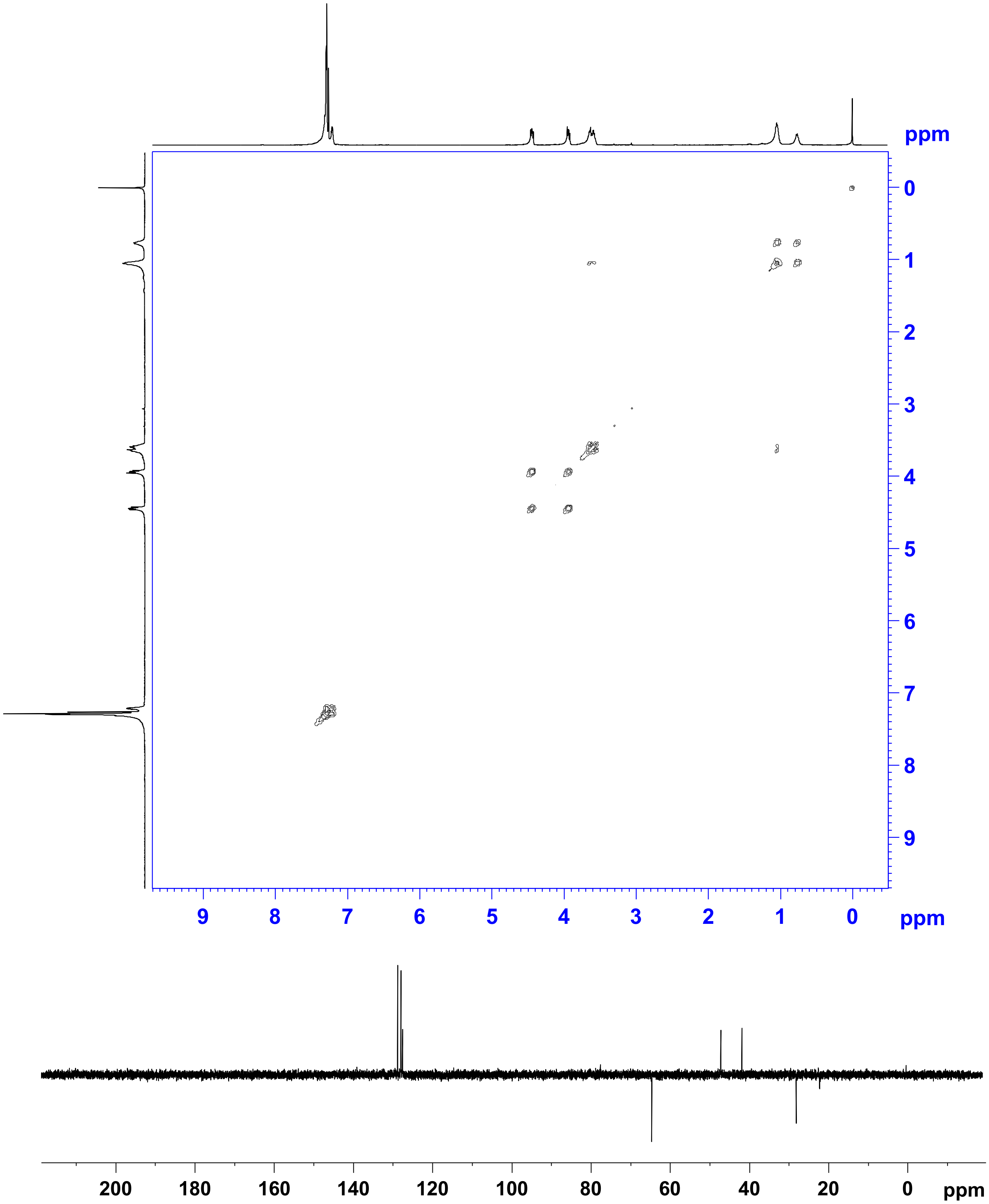

Figure S9 continue. ^{1}H COSY (c) and ^{13}C DEPT 135 (d) NMR spectra of PPAT in $CDCl_3$ at room temperature.

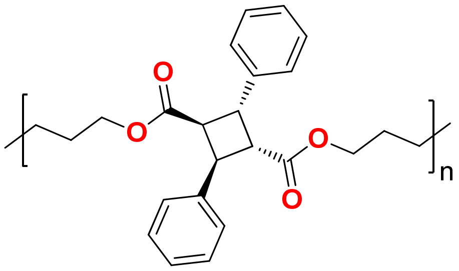

PHAT

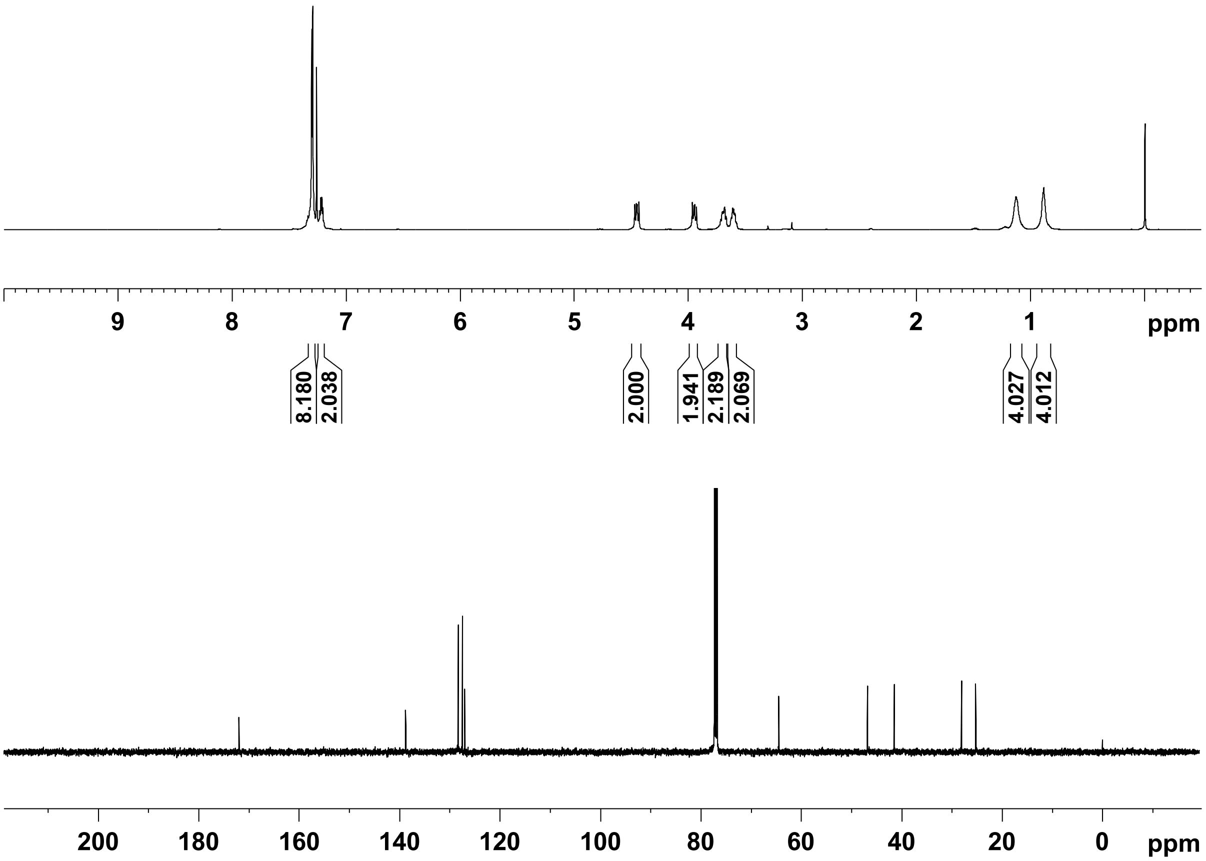

Figure S10. ^{1}H (a) and ^{13}C (b) NMR spectra of PHAT in $CDCl_3$ at room temperature.

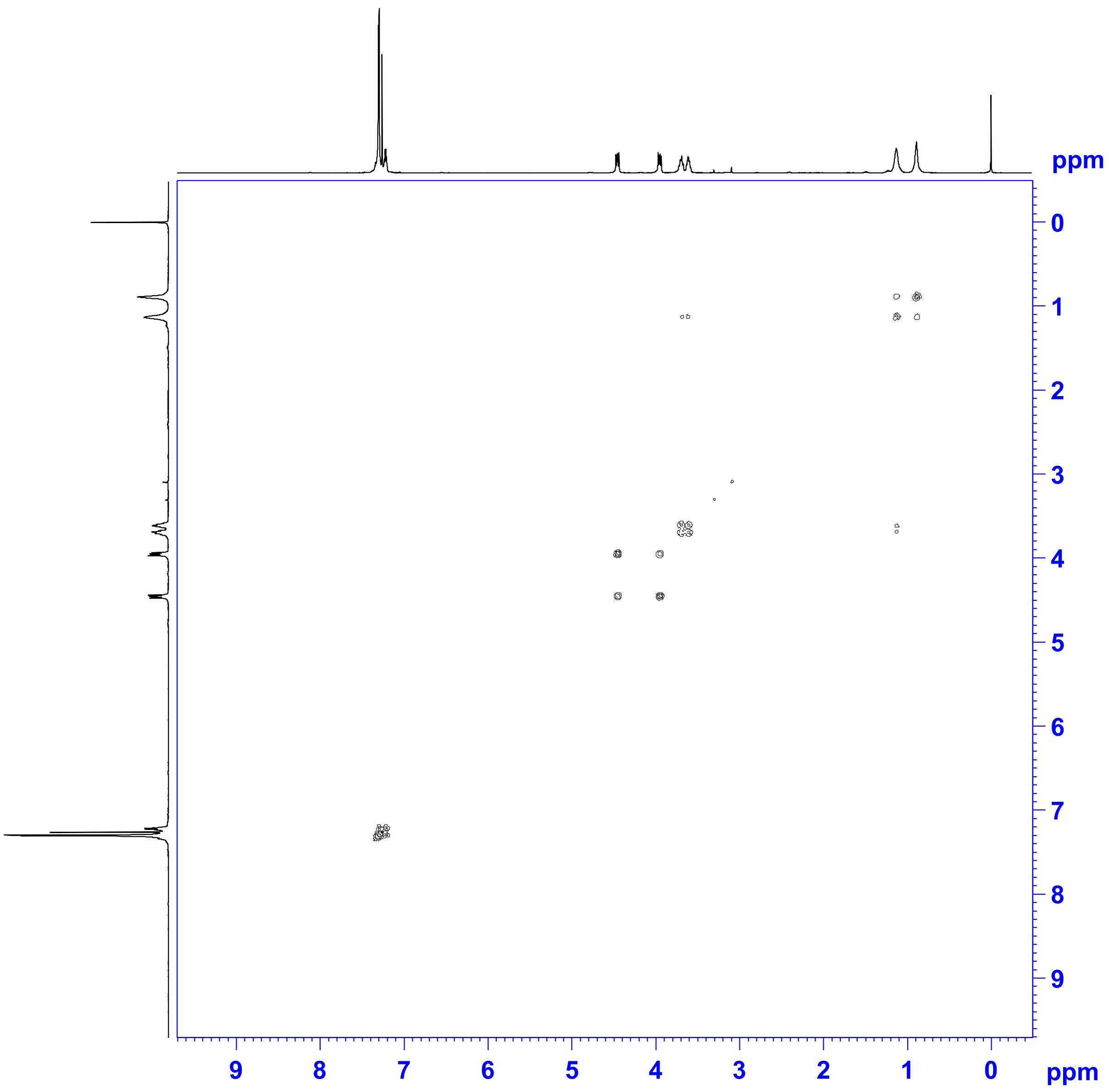

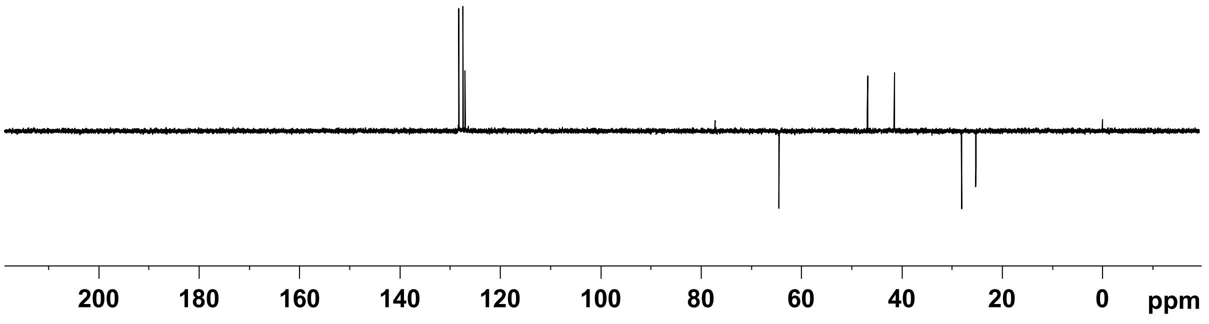

Figure S10 continue. ^{1}H COSY (a) and ^{13}C DEPT 135 (b) NMR spectra of PHAT in $CDCl_3$ at room temperature.

3.4. FT-IR of *α*-Truxillic Acid and Poly-*α*-Truxillates

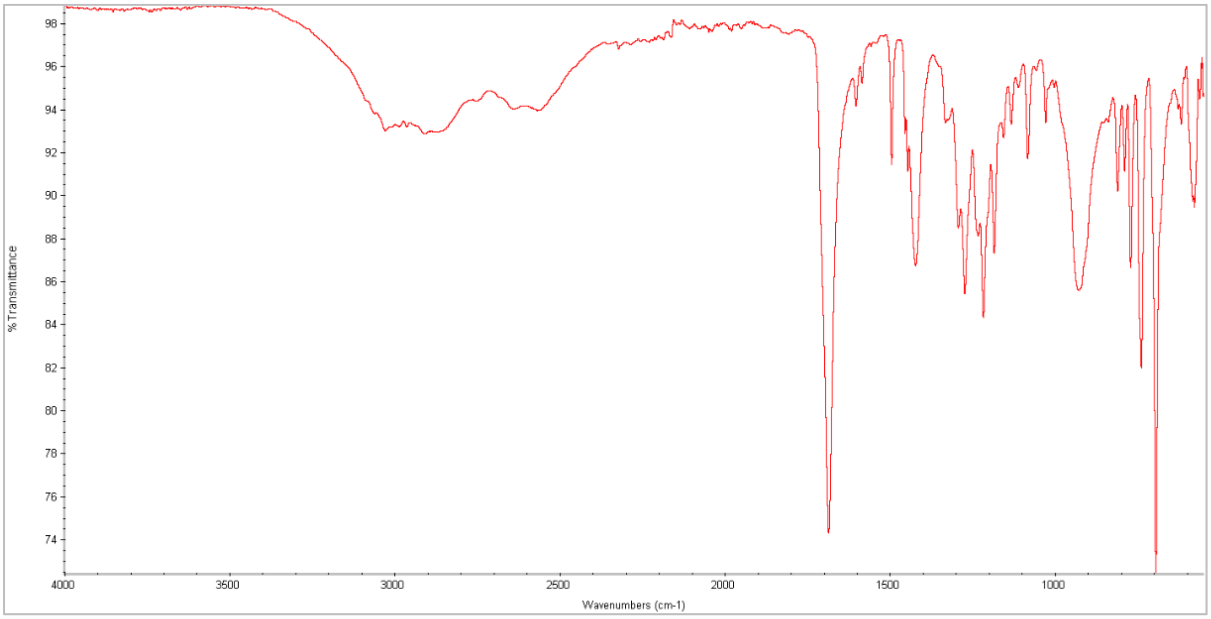

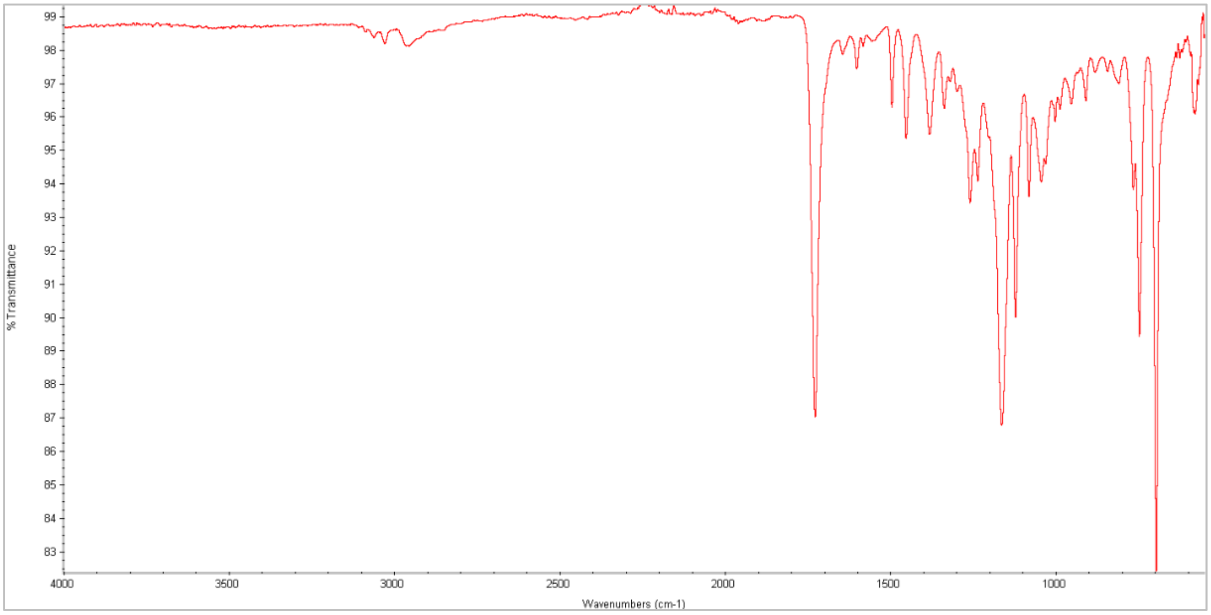

Figure S11. The FT-IR spectra of **CBDA-1** (up, *α*-truxillic acid) and PEAT (down).

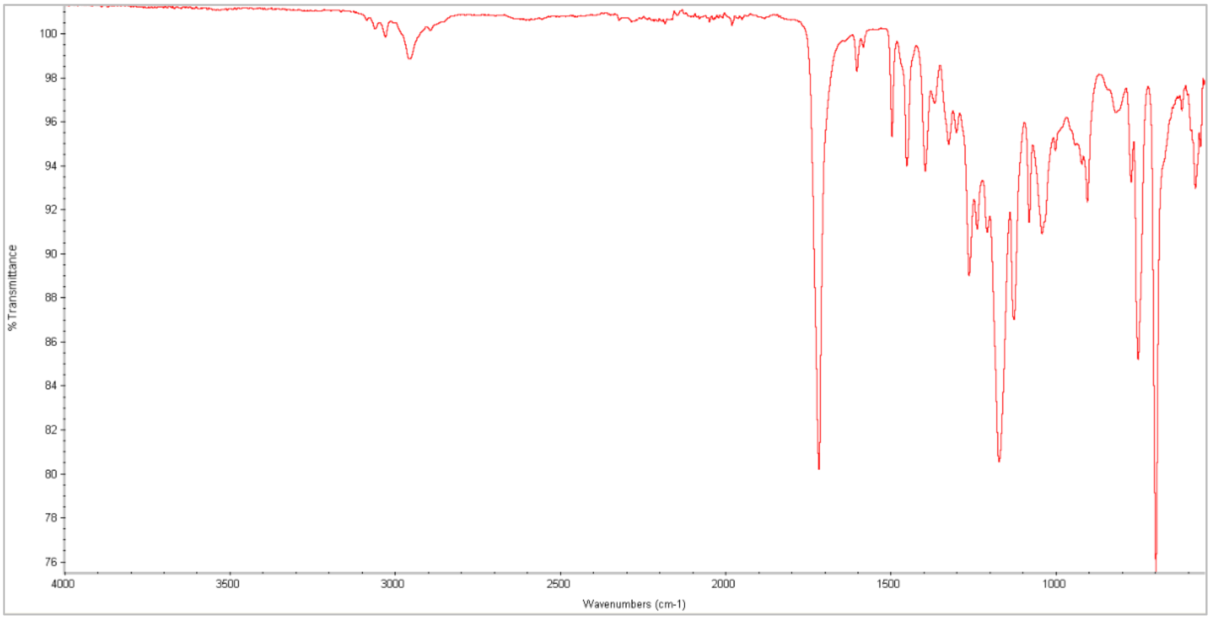

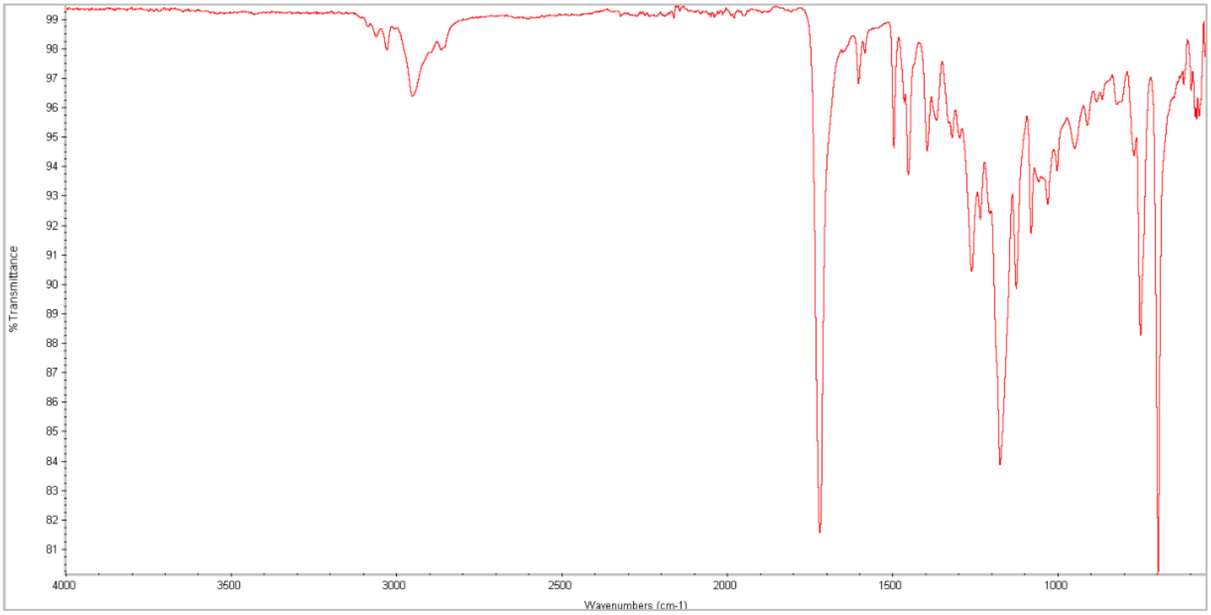

Figure S11 continue. The FT-IR spectra of PBAT (up) and PPAT (down).

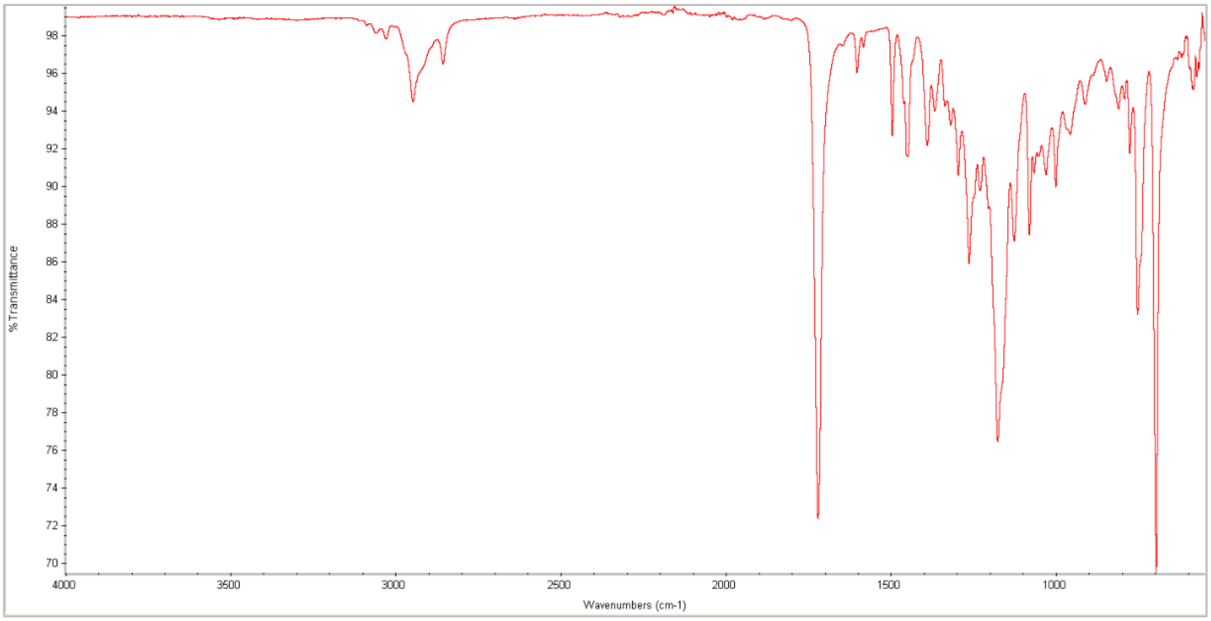

Figure S11 continue. The FT-IR spectrum of PHAT.

3.5. Powder X-ray Diffraction of Poly-α-Truxillates

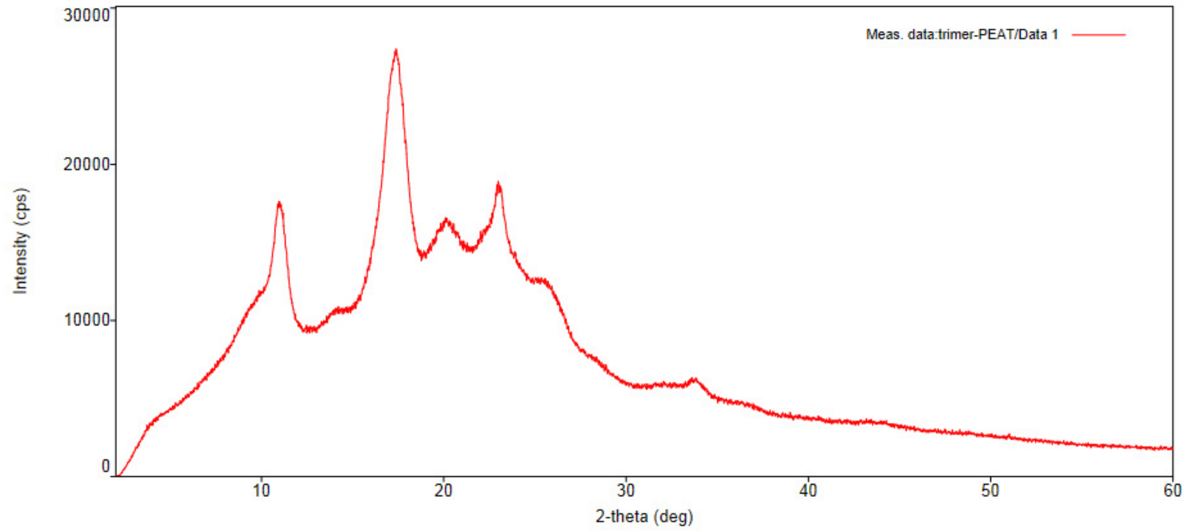

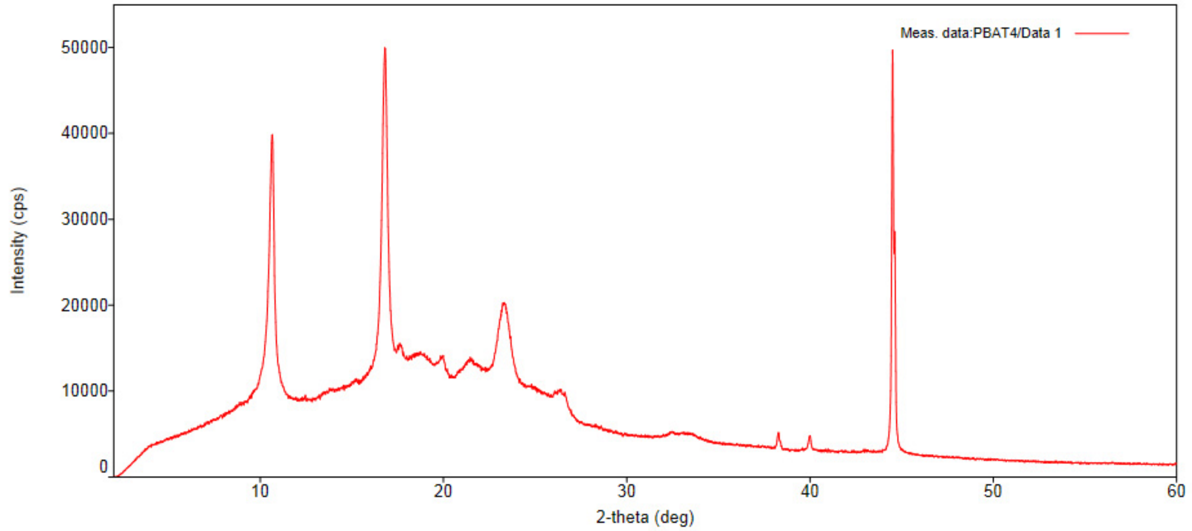

Figure S12. Powder X-ray diffraction (PXRD) patterns of PEAT (up) and PBAT (down). The results indicated a partially crystalline state.

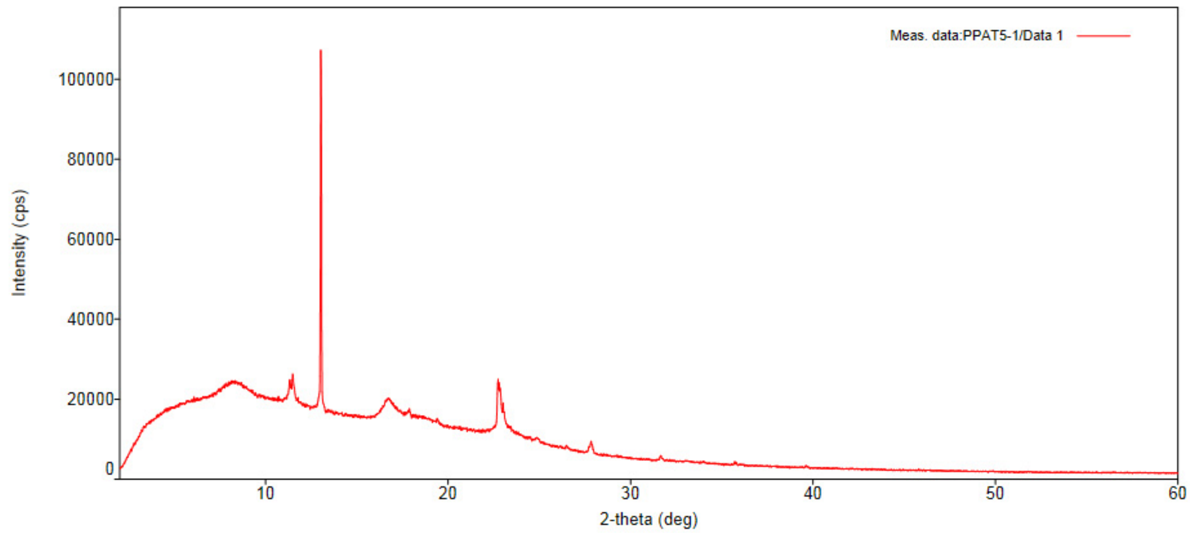

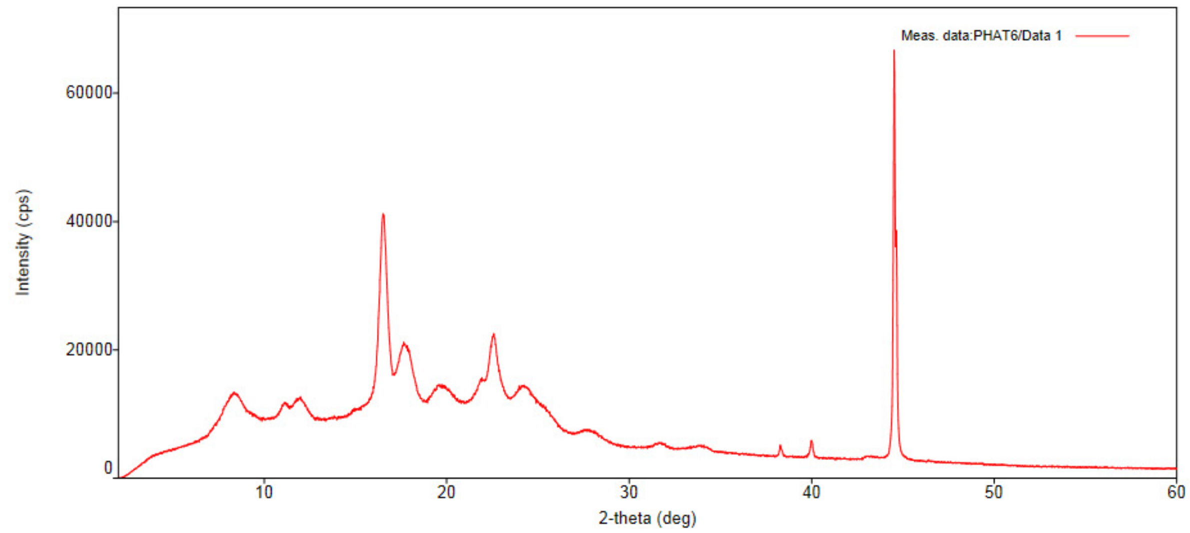

Figure S12 Continue. Powder X-ray diffraction (PXRD) patterns of PPAT (up) and PHAT (down). The results indicated a partially crystalline state.

3.6. MS Spectra

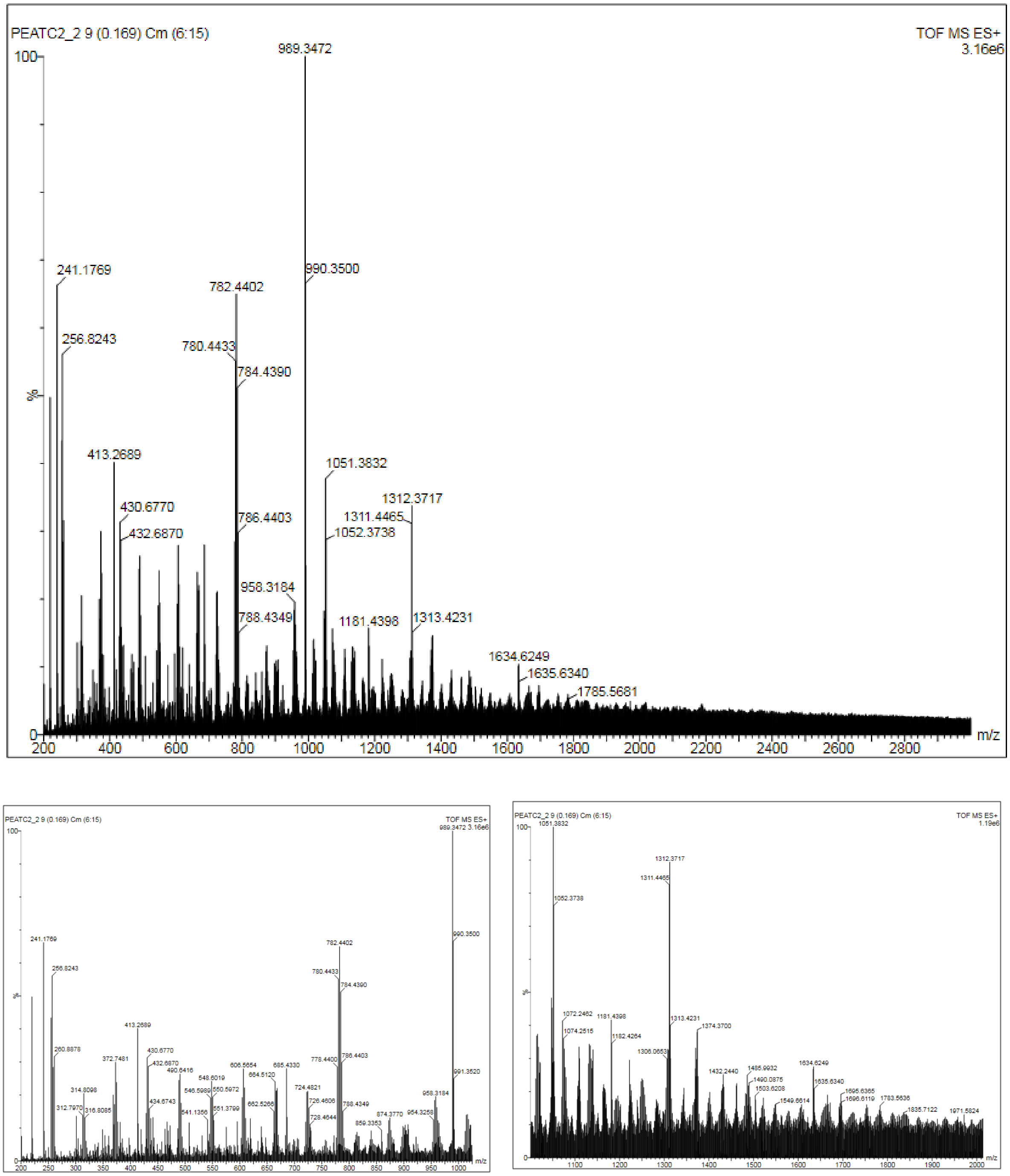

Figure 13. MS spectra of PEAT.

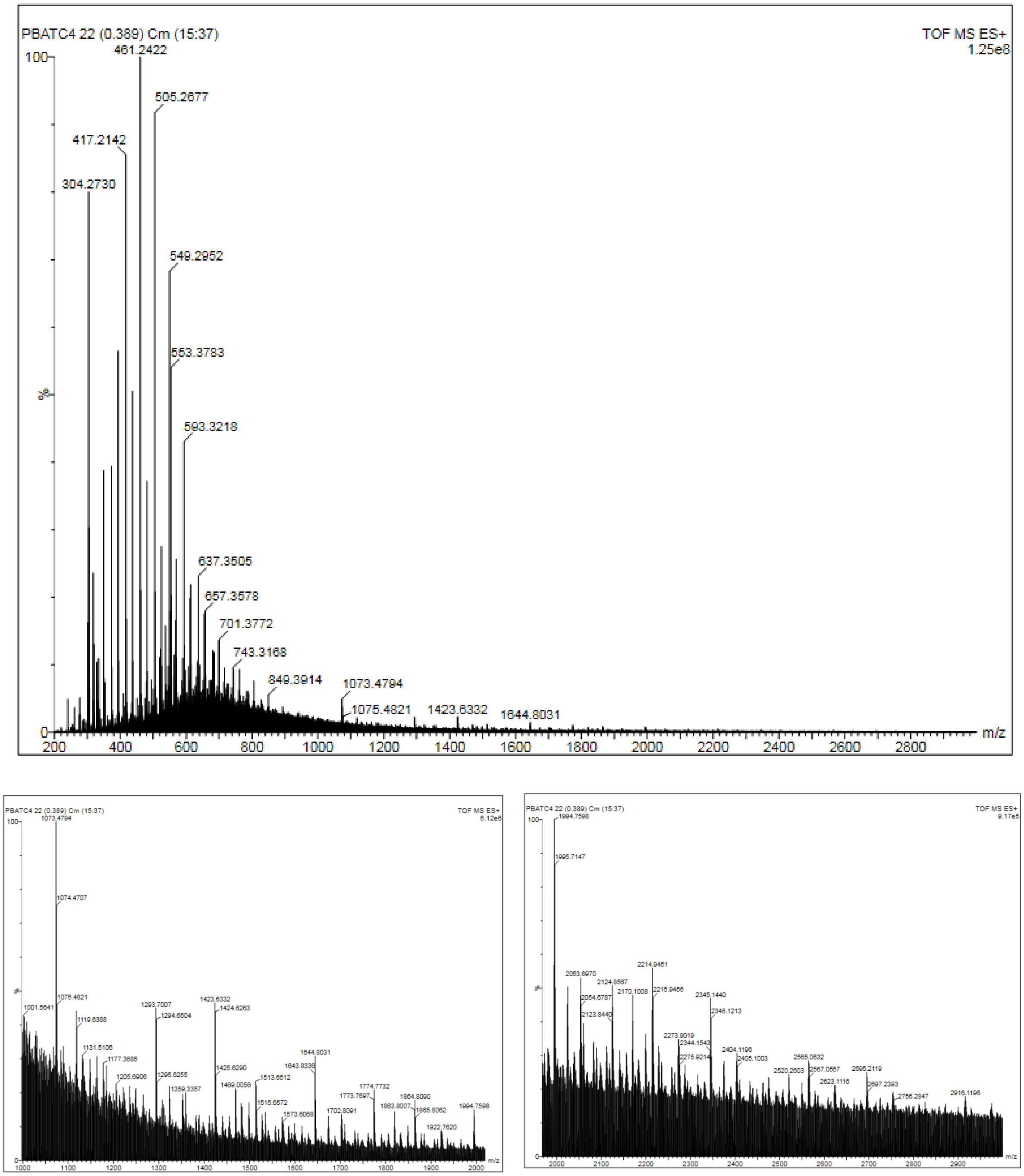

Figure 14. MS spectra of PBAT.

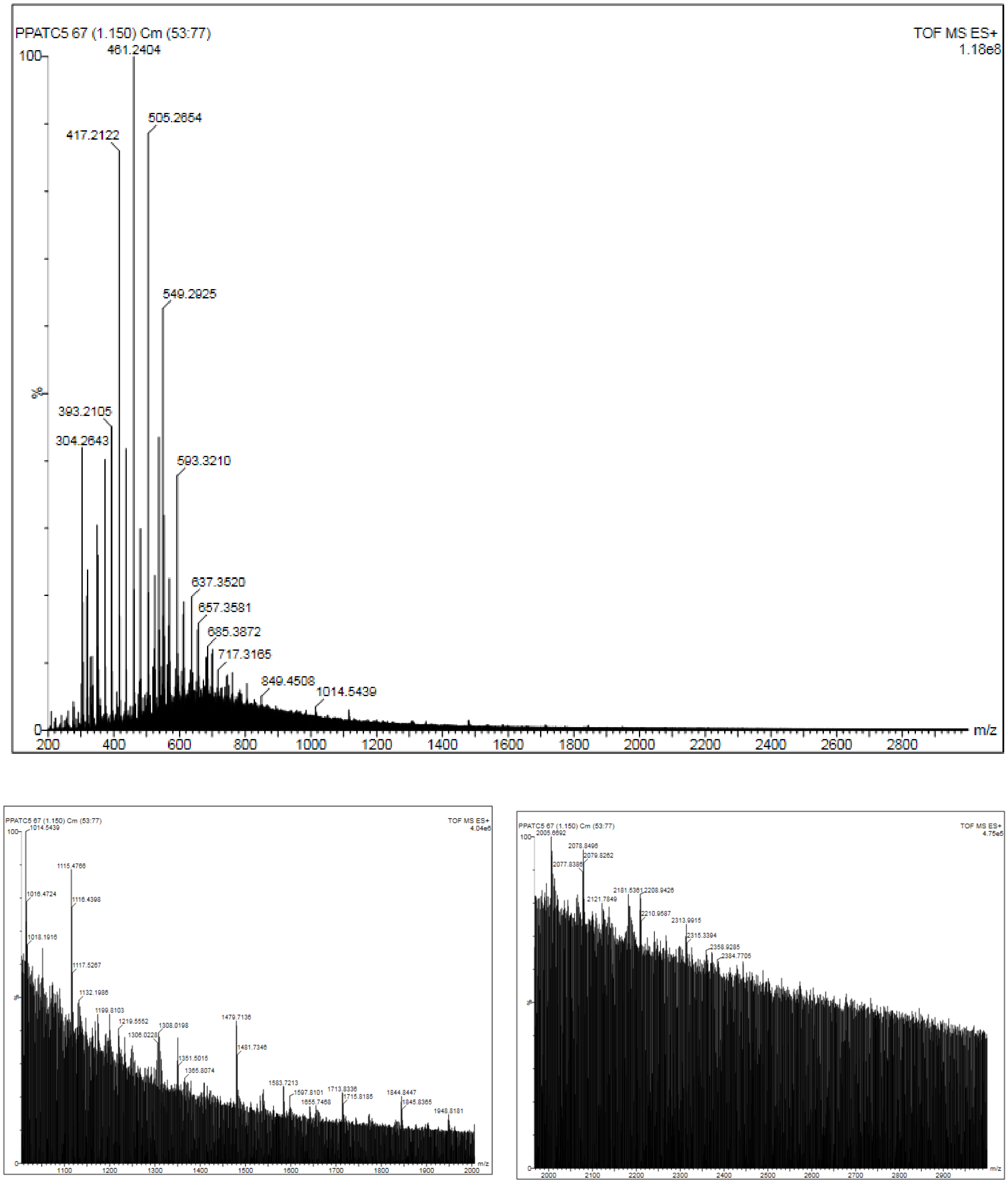

Figure 15. MS spectra of PPAT.

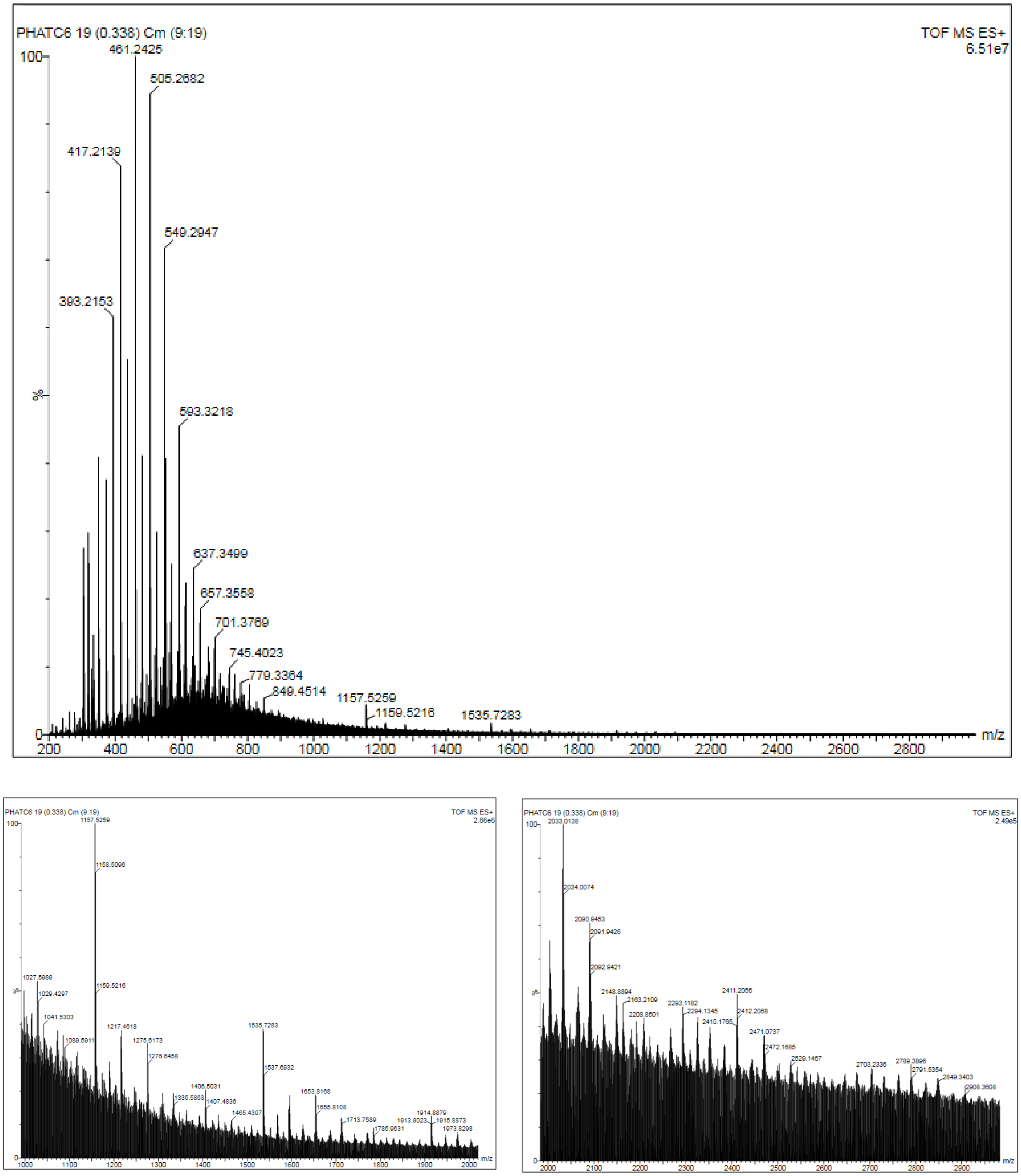

Figure 16. MS spectra of PHAT.

3.7. DSC

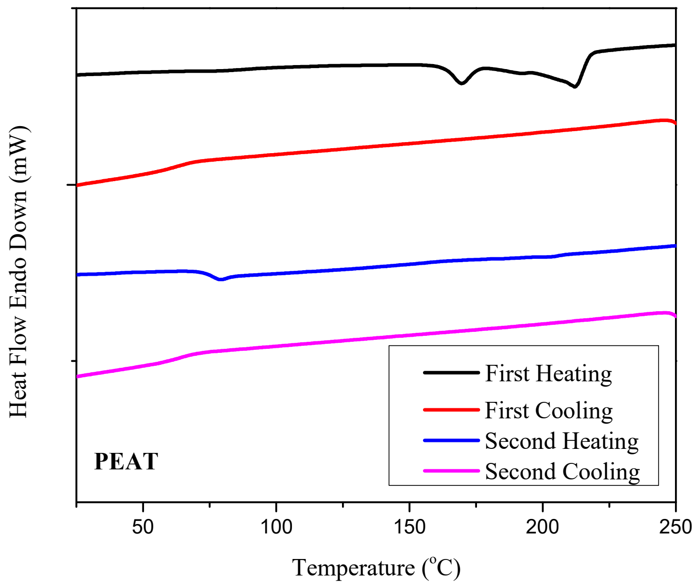

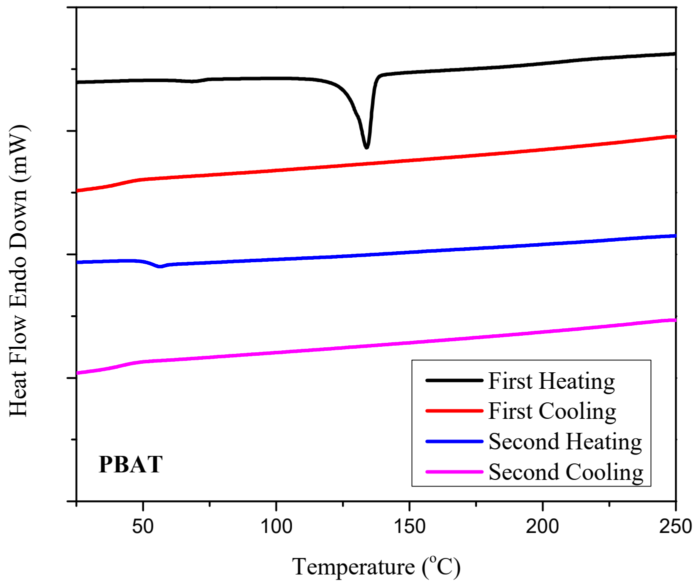

Figure S17. DSC results of PEAT and PBAT.

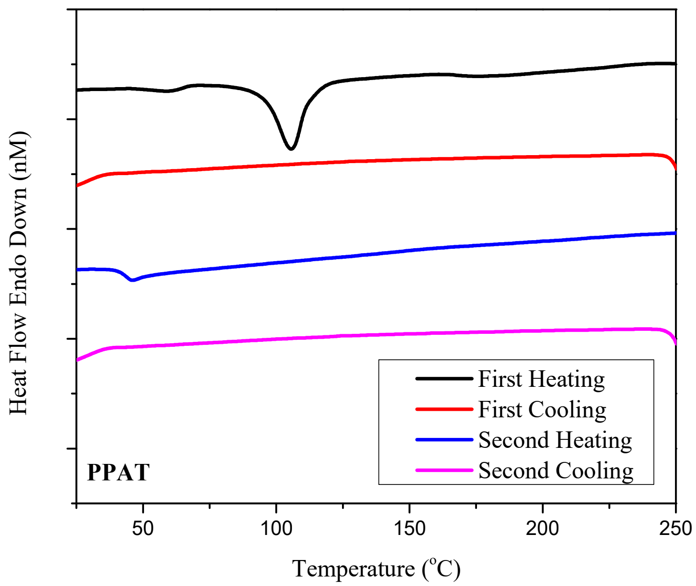

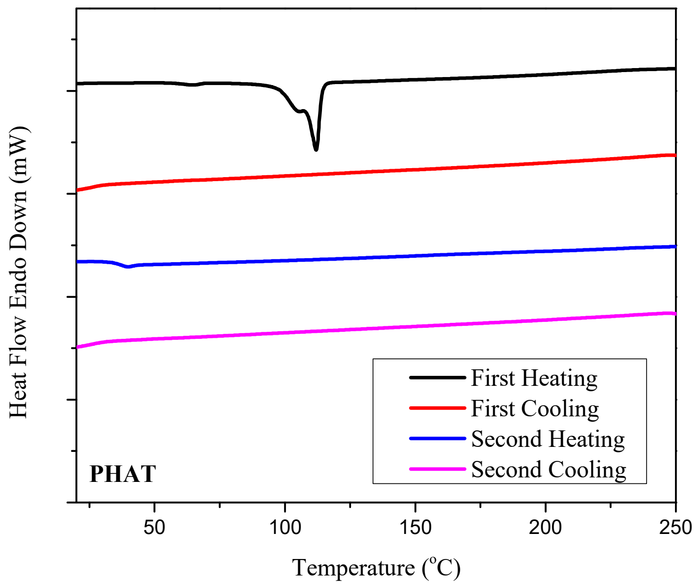

Figure S17 continue. DSC results of PPAT and PHAT.

3.8. TGA

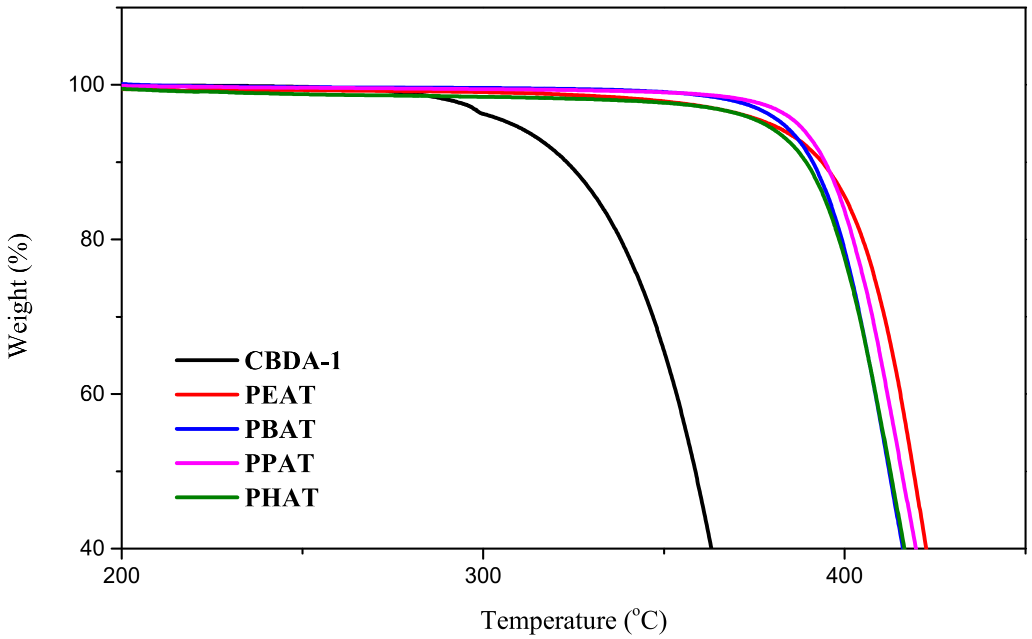

Figure S18. TGA of **CBDA-1** and poly-α-truxillate operated under nitrogen (50.0 ml/min) with rate 20 °C/min. To get a clear view, partial results were magnified. For full scale of TGA results, see the Figure 5 in the manuscript.

3.9. Polydispersity Indices (PDI)

Table S2. PDI of the poly-α-truxillates

Poly-α-truxillates	PEAT	PBAT	PPAT	PHAT
**PDI**	1.88	1.47	1.57	1.85
